# Association of marine PUFAs intakes with cardiovascular disease, all-cause mortality, and cardiovascular mortality in American adult male patients with dyslipidemia: the U.S. National Health and Nutrition Examination Survey, 2001 to 2016

**DOI:** 10.1186/s12937-023-00873-6

**Published:** 2023-10-06

**Authors:** Xuanfeng Tang, Xinyi Lv, Ruohua Wang, Xiaoqing Li, Wenyu Xu, Nan Wang, Shuran Ma, He Huang, Yucun Niu, Xiangju Kong

**Affiliations:** 1 Department of Nutrition and Food Hygiene, School of Public Health, Key Laboratory of Precision nutrition and health, Ministry of Education, Harbin Medical University, Harbin, China; 2 Department of Gynaecology, First Affiliated Hospital of Harbin Medical University, Harbin, People’s Republic of China

**Keywords:** Dyslipidemia, Mortality, Marine PUFAs, CVD, NHANES

## Abstract

**Background:**

The relationship between marine polyunsaturated fatty acid (PUFA) intake and cardiovascular disease and mortality in dyslipidemic patients is unclear. Men with dyslipidemia have a higher risk of cardiovascular disease than women, and PUFA supplementation may be more beneficial in men.

**Objective:**

The purpose of this study was to assess the relationship between different types of marine polyunsaturated fatty acids intakes and cardiovascular disease, all-cause mortality, and cardiovascular mortality in adult U.S. males with dyslipidemia.

**Methods:**

The study ultimately included 11,848 adult men with dyslipidemia who were screened from the National Health and Nutrition Examination Survey (NHANES) between 2001 and 2016. This was linked to the 2019 National Death Index (NDI) records to establish a prospective cohort. In the study, a logistic regression model was established to assess the relationship between PUFA intake and prevalent CVD, and a Cox proportional hazards regression model was established to assess the relationship between PUFA intake and death.

**Results:**

In the fully adjusted models, compared with participants in the lowest tertile, participants with the highest DPA intake were associated with a lower risk of CVD (CVD: OR = 0.71, 95%CI: 0.55, 0.91; angina: OR = 0.54, 95%CI: 0.38, 0.79; stroke: OR = 0.62, 95%CI: 0.43, 0.89), but not with three subtypes of congestive heart failure, coronary heart disease, and myocardial infarction. And the highest tertile level of DPA intake can reduce all-cause mortality (HR = 0.77, 95%CI: 0.64, 0.91) and CVD mortality (HR = 0.68, 95%CI: 0.52, 0.90).

**Conclusions:**

Cardiovascular disease risk, all-cause mortality, and CVD mortality were inversely associated with dietary DPA intake but not EPA and DHA intakes in U.S. male participants with dyslipidemia.

**Supplementary Information:**

The online version contains supplementary material available at 10.1186/s12937-023-00873-6.

## Introduction

Cardiovascular disease (CVD) is currently the leading cause of death and disability in the world and one of the major public health problems contributing to the global burden of significant disease [1]. CVD morbidity and mortality in adults with dyslipidemia in the United States are strongly correlated with diet [2]. Previous studies have shown that dyslipidemia is an important risk factor for coronary artery disease and stroke [3]. There was a gender difference that dyslipidemia was more strongly associated with cardiovascular disease in males [4].

Polyunsaturated fatty acids (PUFAs) such as docosahexaenoic acid (DHA), eicosapentaenoic acid (EPA) and docosapentaenoic acid (DPA) are commonly found in marine plants and animals [5, 6]. Results of a population-based randomized controlled trial suggest that marine PUFAs may have protective effects on the cardiovascular system [7]. Studies have found that DPA is an intermediate product of the metabolism of EPA and DHA in the human body, but due to the low content of DPA in nature and the high price of pure products, in-depth research on it is limited [8, 9]. Current research focuses on DHA and EPA, which can decrease circulating triglyceride levels, reduce inflammation, and inhibit the expression of atherosclerosis-related genes to reduce the risk of cardiovascular disease [10, 11]. However, attributed to the specificity of the studies, the effects of marine PUFAs are inconsistent. The STRENGTH randomized clinical trial did not reduce the risk of CVD in patients with high cardiovascular risk such as dyslipidemia [12]. Contrary results were found in the Icosapent Ethyl-Intervention Trial (Reduce-IT) study, which found that the use of EPA preparations can reduce the risk of CVD events in patients with abnormal blood lipid levels taking statins [13].

Patients with dyslipidemia have a higher risk of cardiovascular disease (CVD) and death. The lowering of blood lipid and inflammation levels of PUFAs may be able to reduce the risk of cardiovascular disease and death in patients with dyslipidemia. And in the past, little research has been done on the relationship between dietary DPA intake levels and the risk of cardiovascular disease in patients with dyslipidemia. Therefore, this study aimed to analyze the association of intake levels of marine PUFAs with cardiovascular disease, all-cause mortality, and cardiovascular disease-specific mortality among American adult males with dyslipidemia.

## Method

The study population was drawn from the National Health and Nutrition Examination Survey (NHANES) database for eight consecutive periods from 2001 to 2016. NHANES is a cross-sectional study of multistage sampling conducted by the National Center for Health Statistics (NCHS) representing the current nutritional and health status of non-institutionalized civilians in states across the country [14]. The NHANES study was approved by the Ethics Review Committee of the National Center for Health Statistics with the written consent of each participant. Detailed survey design, sampling methods, and data are available on the website [15].

### Exclusion and inclusion criteria

**Fig. 1:**
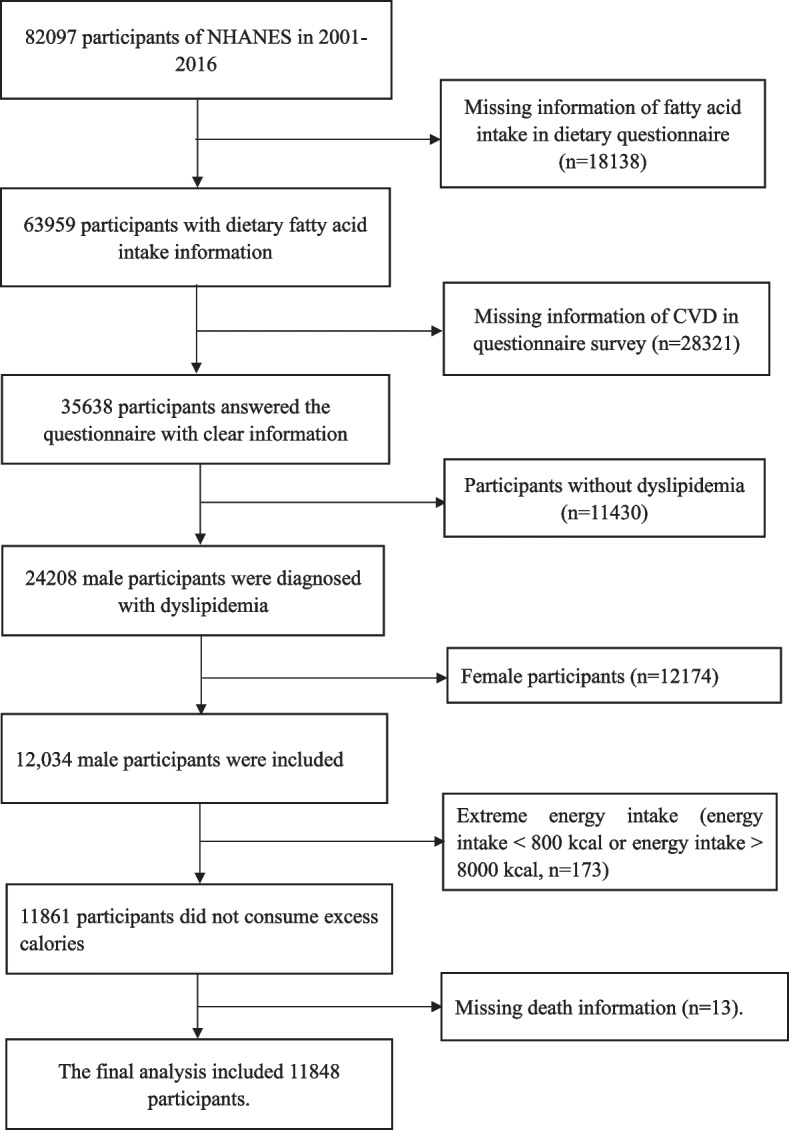
Flow chart of sample selection

Adult males with dyslipidemia were selected from the NHANES database. Dyslipidemia was defined as serum triglyceride level ≥ 150 mg/dl, total cholesterol ≥ 200 mg/ dl, LDL ≥ 150 mg/dl, HDL < 40 mg/dl, or being on cholesterol medication, or being diagnosed with hypercholesterolemia by a physician (https://www.nhlbi.nih.gov/files/docs/guidelines/atglance.pdf). The total population analyzed in the study included 82,097 participants from 2001 to 2016. Participants lacking information on dietary PUFAs intakes (*n* = 18,138), or cardiovascular disease diagnosis (*n* = 28,321), or death(*n* = 13), or normolipidemia (*n* = 11,430), or female with dyslipidemia (*n* = 12,174), or extreme energy intake (energy intake < 800 kcal or energy intake > 8000 kcal, *n* = 173) were excluded and 11,848 male with dyslipidemia were recruited (Fig. 1).

### Exposures

Exposures in this study were obtained by dietary assessment using a 24-h dietary recall survey to obtain food intake discontinuously over 2 days. Dietary data were only collected on the first day during 2001–2002. Second-day dietary data were also collected after an interval of 3–10 days in subsequent survey cycles. Dietary energy and nutrient intakes were estimated using the USDA Dietary Research Food and Nutrition Database. Dietary intake components were integrated into 37 MyPyramid major groups and subgroups according to the USDA MyPyramid Equivalency Database 2.0 (MPED 2.0) User's Guide for Survey Foods. To ensure the comparability of data, two dietary survey data in the seven cycles from 2003 to 2016 were averaged. All interviewers have completed professional training to reduce bias. The exposures in this study were marine PUFAs in the diet, including eicosapentaenoic acid (EPA), docosahexaenoic acid (DHA), and docosapentaenoic acid (DPA).

### Outcomes

The cardiovascular diseases in this study included five categories: congestive heart failure (*n* = 521), coronary heart disease (*n* = 860), angina (*n* = 465), myocardial infarction (*n* = 851), and stroke (*n* = 508). The outcome of the cross-sectional study, cardiovascular disease, was collected by questionnaire, which is a self-reported disease diagnosed by the physician. Prospective cohort study outcome, death status, as determined by the 2019 National Death Index (NDI) as of December 31. NDI is a fairly reliable and widely used resource for death recognition. ICD-10 was used to determine disease-specific death. ICD-10 codes I00-I09, I11, I13, I20-I51, or I60-I69 were assigned death due to CVD. The median follow-up time was 7.83 years. There were 2271 all-cause deaths and 749 cardiovascular deaths.

### Covariates

The covariates in our study included age (< 60 years old or ≥ 60 years old), race/ethnicity (Mexican American/ non-Hispanic white/ non-Hispanic black/ other Hispanic/ other race), education (less than high school/ high school or equivalent/ college or AA Degree/ university or above), marital status (married/ separated/ never married), ratio of family income to poverty (PIR ≤ 1.30, 1.30–1.85, > 1.85), body mass index (BMI, kg/m^2^), physical activity status (yes/no recreational moderate and vigorous physical activity in the past week), smoking status (yes/no smoking 100 cigarettes in a lifetime up to date), alcohol consumption status (yes/no drinking at least 12 alcoholic beverages per year), total energy intake over 24 h of dietary recall (kcal), total fat intake (g), total cholesterol intake (g), as well as hypertension, diabetes, cancer, and osteoarthritis were self-reported disease which were diagnosed by the physician.

### Statistical methods

Because the NHANES study was conducted based on complex sampling procedures, weighted analysis was used for the results of this study. The data in this study represented a national population of approximately 77 million. The logistic regression and Cox proportional hazard regression models were established for disease and death, respectively. Odds ratio (OR) and 95%CI were used for logistic multi-factor regression model results, while hazard ratio (HR) and 95%CI were used for Cox proportional hazard regression model results. Survival times in the Cox proportional risk regression model were calculated based on the time of follow-up between the date of the interview and the participant's death or the end of 2019. Dietary marine PUFAs were divided into tertiles and the lowest tertile group was used as a reference group. In the full logistic multi-factor regression model, we adjusted for age, race, education, marital status, family poverty, BMI, physical activity, smoking status, alcohol consumption, hypertension, diabetes, total energy intake, total fat intake, and total cholesterol intake. In the final Cox proportional hazard regression model, we adjusted for age, race, education, marital status, family poverty, BMI, physical activity, smoking, alcohol consumption, hypertension, diabetes, cancer, osteoarthritis, total energy intake, total fat intake, and total cholesterol intake. All continuous variables were measured by Wilcox test and expressed as median and interquartile range (Median ± IQR), while categorical variables were measured by Wald test and expressed as percentages. All statistical analyses were performed using R 4.2.2 software, and bilateral *p* < 0.05 was considered statistically significant.

### Sensitivity analysis

In addition, six sensitivity analyses were conducted in this study. In the first group, we analyzed the association of marine PUFAs intake with cardiovascular disease, all-cause mortality, and cardiovascular mortality in obese or overweight populations (BMI > 27). Participants who had no extreme dietary fat intake and were followed for more than 2 years were in the second and third groups. In the fourth group, we analyzed the association of marine PUFAs intake with cardiovascular disease, all-cause mortality, and cardiovascular mortality in elderly men with dyslipidemia who were over 60 years old. In the last two sets of sensitivity analyses, we selected women with dyslipidemia and all male participants who met the criteria, respectively.

## Result

### Baseline characteristics

Baseline information for participants was shown in Table 1. Participants in the CVD, all-cause mortality, and CVD mortality groups were likely to be older, non-hispanic, divorced, lower ratio of family income to poverty, high school education or less, and high prevalence of hypertension, diabetes, cancer, osteoarthritis, and poor smoking, physical activity (all *p* < 0.05). In addition, dietary total energy, total fat, total cholesterol, DPA and EPA intakes in the CVD, all-cause mortality and CVD mortality groups were significantly lower than the whole group (all *p* < 0.05).

**Table 1:** Baseline characteristics of the participants

Characteristic	Overall *N* = 11,848 ^1^	CVD prevalence *N* = 1790 ^1^	*P*-value ^2^	ALL-cause mortality *N* = 2271 ^1^	*P*-value ^2^	CVD mortality *N* = 749 ^1^	*P*-value ^2^
**Age**			< 0.001		< 0.001		< 0.001
< 60	7,619 (75.6%)	438 (33.7%)		491 (36.2%)		124 (28.2%)	
> = 60	4,229 (24.4%)	1,352 (66.3%)		1,780 (63.8%)		625 (71.8%)	
**BMI**			< 0.001		0.090		0.017
< 30	7,338 (61.6%)	1,016 (53.8%)		1,425 (59.2%)		457 (55.3%)	
> = 30	4,510 (38.4%)	774 (46.2%)		846 (40.8%)		292 (44.7%)	
**Race**			< 0.001		< 0.001		< 0.001
Mexican American	2,025 (8.5%)	178 (3.9%)		230 (3.4%)		72 (2.9%)	
Non-Hispanic Black	2,060 (8.4%)	318 (8.9%)		375 (8.9%)		125 (8.9%)	
Non-Hispanic White	5,968 (72.6%)	1,126 (81.2%)		1,501 (81.6%)		503 (82.1%)	
Other Hispanic	932 (4.9%)	96 (2.3%)		101 (3.3%)		28 (3.2%)	
Other Race	863 (5.7%)	72 (3.6%)		64 (2.7%)		21 (2.9%)	
**Education**			< 0.001		< 0.001		< 0.001
Less than 12th grade	3,116 (16.5%)	573 (25.7%)		793 (26.9%)		262 (26.6%)	
GED or equivalent	2,824 (23.9%)	433 (25.8%)		585 (28.1%)		189 (27.8%)	
College or AA degree	3,120 (29.6%)	439 (25.5%)		505 (25.8%)		166 (25.2%)	
College or above	2,788 (30.0%)	345 (23.0%)		388 (19.2%)		132 (20.4%)	
**Marriage**			< 0.001		< 0.001		< 0.001
Married	8,443 (71.4%)	1,240 (72.5%)		1,535 (68.3%)		515 (69.0%)	
Never married	1,558 (14.9%)	103 (6.5%)		149 (8.2%)		45 (6.3%)	
Separated	1,847 (13.7%)	447 (20.9%)		587 (23.5%)		189 (24.7%)	
**PIR**			< 0.001		< 0.001		< 0.001
< = 1.30	2,994 (16.7%)	526 (21.2%)		655 (20.4%)		217 (20.4%)	
1.30 ~ 1.85	1,400 (9.1%)	238 (11.2%)		338 (13.3%)		119 (14.3%)	
> 1.85	7,454 (74.2%)	1,026 (67.7%)		1,278 (66.2%)		413 (65.3%)	
**Smoke**	6,802 (54.8%)	1,277 (72.1%)	< 0.001	1,638 (73.1%)	< 0.001	506 (68.0%)	< 0.001
**Drink**	9,981 (85.5%)	1,474 (84.3%)	0.380	1,829 (80.9%)	< 0.001	587 (78.1%)	< 0.001
**Sport**	6,242 (60.1%)	705 (44.4%)	< 0.001	890 (45.4%)	< 0.001	288 (46.0%)	< 0.001
**Hypertension**	4,668 (34.7%)	1,314 (69.3%)	< 0.001	1,340 (55.2%)	< 0.001	471 (61.3%)	< 0.001
**Diabetes**	2,024 (12.8%)	619 (30.1%)	< 0.001	660 (26.4%)	< 0.001	226 (28.8%)	< 0.001
**Arthritis**	972 (8.2%)	273 (17.2%)	< 0.001	303 (14.6%)	< 0.001	116 (16.2%)	< 0.001
**Cancer**	1,268 (10.1%)	399 (22.7%)	< 0.001	555 (23.9%)	< 0.001	152 (21.6%)	< 0.001
**Total energy intake (kcal)**	2,334.0 (1,837.5,2,955.6)	2,021.3 (1,627.8,2,545.4)	< 0.001	2,020.2 (1,583.9,2,536.2)	< 0.001	1,992.5 (1,562.0,2,494.4)	< 0.001
**Total fat intake (g)**	87.1 (64.2,117.6)	77.7 (56.4,101.1)	< 0.001	76.4 (56.0,102.3)	< 0.001	73.7 (55.1,98.3)	< 0.001
**Total cholesterol intake (g)**	294.0 (187.5,442.1)	267.4 (166.3,408.1)	< 0.001	276.5 (161.5,405.0)	< 0.001	271.0 (154.0,415.4)	0.004
**DPA (mg)**	15.0 (4.0,29.0)	12.0 (3.0,24.0)	< 0.001	9.0 (0.0,22.0)	< 0.001	9.0 (0.0,21.5)	< 0.001
**EPA (mg)**	9.0 (3.5,22.0)	7.5 (3.0,19.5)	0.079	6.5 (2.5,18.5)	< 0.001	6.0 (2.0,17.5)	< 0.001
**DHA (mg)**	30.0 (7.5,75.0)	27.4 (7.0,66.5)	0.150	28.0 (7.0,70.0)	0.210	27.0 (5.4,64.0)	0.110

CVD prevalence including: congestive heart failure (*n* = 521), coronary heart disease (*n* = 860), angina (*n* = 465), myocardial infarction (*n* = 851) and stroke (*n* = 508)

*BMI *body mass index, *PIR *ratio of family income to poverty, *DPA *docosapentaenoic acid, *EPA *eicosapentaenoic acid, *DHA* hexadecapentaenoic acid

^1^n (unweighted) (%); Median (25%,75%)

^2^Wald test of independence for complex survey samples; Wilcoxon rank-sum test for complex survey samples

### Marine PUFAs and CVD

The association between dietary intake of marine PUFAs and cardiovascular disease were represented by OR and 95%CI (Tables 2, 3 and 4). In the multi-factor adjusted logistic regression model 3, compared with participants in the lowest tertile, participants with the highest DPA intake were associated with a lower risk of CVD (CVD: OR = 0.71, 95%CI: 0.55, 0.91; angina: OR = 0.54, 95%CI: 0.38, 0.79; stroke: OR = 0.62, 95%CI: 0.43, 0.89). However, DPA intake was associated with coronary heart disease (OR = 0.73, 95%CI: 0.56–0.95) only in logistic regression model 1. DPA intake was not associated with the risk of congestive heart failure and myocardial infarction in all models. DHA and EPA intake were not associated with total CVD in all models. But in the model without full adjustment, participants in the highest tertile of DHA intake were associated with a lower risk of stroke (OR = 0.71, 95%CI: 0.51, 0.99) compared with the lowest group. The highest tertile of EPA intake was associated with a lower risk of stroke (OR = 0.62, 95%CI: 0.44, 0.87) among participants. But in the fully adjusted model, no association was found between EPA and DHA intake and the five cardiovascular diseases.

**Table 2:** Associations of the DPA intake with cardiovascular disease

	**Model 1**			**Model 2**			**Model 3**		
	**OR**^a^	**95% CI**^a^	***p*-value**	**OR**^a^	**95% CI**^a^	***p*-value**	**OR**^a^	**95% CI**^a^	***p*-value**
**CVD**									
**Q1**	Ref	Ref		Ref	Ref		Ref	Ref	
**Q2**	0.86	0.70, 1.04	0.125	0.87	0.72, 1.07	0.182	0.85	0.69, 1.05	0.128
**Q3**	0.69	0.56, 0.87	0.001**	0.71	0.56, 0.90	0.006**	0.71	0.55, 0.91	0.007**
**CHF**									
**Q1**	Ref	Ref		Ref	Ref		Ref	Ref	
**Q2**	0.88	0.66, 1.18	0.384	0.92	0.68, 1.24	0.584	0.88	0.63, 1.21	0.424
**Q3**	0.86	0.62, 1.21	0.393	0.91	0.64, 1.30	0.599	0.88	0.59, 1.31	0.529
**CHD**									
**Q1**	Ref	Ref		Ref	Ref		Ref	Ref	
**Q2**	0.84	0.66, 1.07	0.161	0.84	0.66, 1.07	0.168	0.85	0.66, 1.09	0.207
**Q3**	0.75	0.58, 0.98	0.036*	0.77	0.58, 1.01	0.058	0.84	0.61, 1.14	0.256
**Angina**									
**Q1**	Ref	Ref		Ref	Ref		Ref	Ref	
**Q2**	0.72	0.51, 1.01	0.057	0.72	0.51, 1.02	0.067	0.70	0.50, 1.00	0.049*
**Q3**	0.56	0.39, 0.80	0.002**	0.57	0.39, 0.83	0.004**	0.54	0.38, 0.79	0.001**
**MI**									
**Q1**	Ref	Ref		Ref	Ref		Ref	Ref	
**Q2**	0.91	0.70, 1.19	0.492	0.94	0.72, 1.23	0.641	0.92	0.71, 1.21	0.554
**Q3**	0.77	0.58, 1.01	0.062	0.80	0.59, 1.08	0.136	0.78	0.57, 1.06	0.111
**Stroke**									
**Q1**	Ref	Ref		Ref	Ref		Ref	Ref	
**Q2**	0.86	0.62, 1.20	0.371	0.88	0.63, 1.24	0.474	0.85	0.60, 1.20	0.347
**Q3**	0.59	0.42, 0.82	0.002**	0.61	0.43, 0.85	0.004**	0.62	0.43, 0.89	0.010*

^*^p < 0.05; **p < 0.01

^a^*OR* odds ratio, *CI *confidence interval, *CHF *congestive heart failure, *CHD *coronary heart disease, *MI *myocardial infarction; multivariate adjusted Logistic regression models of DPA intake with cardiovascular disease: Model 1: Age and race; Model 2: Model 1 + education, Marital status, ratio of family income to poverty, BMI, physical activity, smoking status and alcohol consumption; Model 3: Model 2 + hypertension, diabetes, cancer, osteoarthritis, total energy intake, total fat intake, and total cholesterol intake

**Table 3:** Associations of the DHA intake with cardiovascular disease

	**Model 1**			**Model 2**			**Model 3**		
	**OR**^a^	**95% CI**	***p*-value**	**OR**^a^	**95% CI**	***p*-value**	**OR**^a^	**95% CI**	***p*-value**
**CVD**									
**Q1**	Ref	Ref		Ref	Ref		Ref	Ref	
**Q2**	0.91	0.74, 1.12	0.376	0.92	0.74, 1.14	0.449	0.97	0.75, 1.25	0.808
**Q3**	0.88	0.72, 1.08	0.211	0.91	0.73, 1.13	0.394	0.98	0.76, 1.26	0.862
**CHF**									
**Q1**	Ref	Ref		Ref	Ref		Ref	Ref	
**Q2**	1.07	0.79, 1.46	0.657	1.10	0.81, 1.48	0.542	1.10	0.78, 1.56	0.571
**Q3**	0.98	0.70, 1.39	0.930	1.02	0.71, 1.46	0.912	1.03	0.67, 1.59	0.900
**CHD**									
**Q1**	Ref	Ref		Ref	Ref		Ref	Ref	
**Q2**	0.81	0.62, 1.05	0.113	0.81	0.62, 1.05	0.111	0.90	0.67, 1.22	0.499
**Q3**	0.88	0.69, 1.13	0.310	0.89	0.69, 1.16	0.386	1.05	0.77, 1.42	0.768
**Angina**									
**Q1**	Ref	Ref		Ref	Ref		Ref	Ref	
**Q2**	0.80	0.58, 1.12	0.198	0.81	0.58, 1.14	0.227	0.88	0.61, 1.27	0.484
**Q3**	0.99	0.69, 1.43	0.976	1.04	0.70, 1.52	0.858	1.12	0.75, 1.68	0.578
**MI**									
**Q1**	Ref	Ref		Ref	Ref		Ref	Ref	
**Q2**	1.01	0.81, 1.26	0.920	1.02	0.81, 1.29	0.846	1.00	0.76, 1.30	0.988
**Q3**	0.99	0.78, 1.27	0.965	1.03	0.78, 1.34	0.852	0.98	0.72, 1.34	0.918
**Stroke**									
**Q1**	Ref	Ref		Ref	Ref		Ref	Ref	
**Q2**	0.89	0.65, 1.21	0.443	0.90	0.65, 1.25	0.526	0.91	0.63, 1.33	0.629
**Q3**	0.71	0.51, 0.99	0.041*	0.73	0.52, 1.03	0.069	0.76	0.51, 1.15	0.190

^*^p < 0.05

^a^*OR *odds ratio, *CI *confidence interval, *CHF *congestive heart failure, *CHD *coronary heart disease, *MI *myocardial infarction; multivariate adjusted Logistic regression models of DHA intake with cardiovascular disease: Model 1:Age and race; Model 2: Model 1 + education, Marital status, ratio of family income to poverty, BMI, physical activity, smoking status and alcohol consumption; Model 3: Model 2 + hypertension, diabetes, cancer, osteoarthritis, total energy intake, total fat intake, and total cholesterol intake

**Table 4:** Associations of the EPA intake with cardiovascular disease

	**Model 1**			**Model 2**			**Model 3**		
	**OR**^a^	**95% CI**^a^	***p*-value**	**OR**^a^	**95% CI**^a^	***p*-value**	**OR**^a^	**95% CI**^a^	***p*-value**
**CVD**									
**Q1**	Ref	Ref		Ref	Ref		Ref	Ref	
**Q2**	1.01	0.82, 1.25	0.921	1.05	0.85, 1.31	0.631	1.07	0.85, 1.35	0.576
**Q3**	0.84	0.69, 1.04	0.111	0.94	0.75, 1.17	0.564	0.98	0.77, 1.25	0.871
**CHF**									
**Q1**	Ref	Ref		Ref	Ref		Ref	Ref	
**Q2**	1.17	0.89, 1.53	0.251	1.25	0.95, 1.65	0.113	1.24	0.94, 1.63	0.135
**Q3**	0.88	0.63, 1.23	0.442	1.00	0.70, 1.42	0.999	1.00	0.71, 1.42	0.980
**CHD**									
**Q1**	Ref	Ref		Ref	Ref		Ref	Ref	
**Q2**	0.93	0.71, 1.21	0.575	0.94	0.72, 1.23	0.671	1.00	0.76, 1.32	0.994
**Q3**	0.97	0.76, 1.24	0.823	1.04	0.81, 1.34	0.741	1.17	0.89, 1.54	0.249
**Angina**									
**Q1**	Ref	Ref		Ref	Ref		Ref	Ref	
**Q2**	0.96	0.69, 1.34	0.818	1.01	0.71, 1.43	0.971	1.03	0.72, 1.47	0.883
**Q3**	0.87	0.63, 1.22	0.425	0.98	0.70, 1.39	0.928	1.03	0.71, 1.51	0.868
**MI**									
**Q1**	Ref	Ref		Ref	Ref		Ref	Ref	
**Q2**	1.04	0.82, 1.31	0.753	1.09	0.86, 1.38	0.469	1.10	0.87, 1.39	0.422
**Q3**	0.82	0.63, 1.05	0.112	0.90	0.69, 1.18	0.462	0.92	0.69, 1.21	0.535
**Stroke**									
**Q1**	Ref	Ref		Ref	Ref		Ref	Ref	
**Q2**	0.77	0.56, 1.06	0.103	0.79	0.58, 1.09	0.155	0.80	0.56, 1.14	0.213
**Q3**	0.62	0.44, 0.87	0.006**	0.69	0.49, 0.96	0.028*	0.72	0.50, 1.04	0.080

^*^p < 0.05; **p < 0.01

^a^*OR *odds ratio, *CI *confidence interval, *CHF *congestive heart failure, *CHD *coronary heart disease, *MI *myocardial infarction; multivariate adjusted Logistic regression models of EPA intake with cardiovascular disease: Model 1:Age and race; Model 2: Model 1 + education, Marital status, ratio of family income to poverty, BMI, physical activity, smoking status and alcohol consumption; Model 3: Model 2 + hypertension, diabetes, cancer, osteoarthritis, total energy intake, total fat intake, and total cholesterol intake

### Marine PUFAs intake with all-cause mortality and CVD mortality

Three Cox proportional hazard regression models in Table 5 show the association of Marine PUFAs intake with all-cause mortality and cardiovascular mortality in adult men with dyslipidemia. The results were shown as HR and 95%CI. The risk of all-cause death among participants with the highest tertile dietary DPA in Cox proportional hazard regression Model 3 (HR = 0.77, 95%CI: 0.64, 0.91) and cardiovascular causes of death (HR = 0.68, 95%CI: 0.52, 0.90) were lower than the lowest tertile. Dietary DHA intake levels were not associated with all-cause mortality or cardiovascular disease-specific mortality in the study population in all models. In Cox proportional hazard Regression Model 1, participants who consumed the highest tertile of EPA had a reduced risk of all-cause death (HR = 0.84, 95%CI: 0.71, 0.99) compared with participants with the lowest tertile. In the fully adjusted model, EPA intake levels were not associated with all-cause mortality and CVD mortality.

**Table 5:** Associations of marine PUFAs intake with all-cause mortality and CVD mortality

	**Model 1**			**Model 2**			**Model 3**		
	**HR**^a^	**95% CI**^a^	***p*-value**	**HR**^a^	**95% CI**^a^	***p*-value**	**HR**^a^	**95% CI**^a^	***p*-value**
	**DPA**								
**ALL-cause**									
**Q1**	Ref	Ref		Ref	Ref		Ref	Ref	
**Q2**	0.84	0.71, 0.99	0.040*	0.88	0.75, 1.03	0.117	0.87	0.74, 1.02	0.088
**Q3**	0.73	0.62, 0.86	< 0.001**	0.76	0.64, 0.91	0.002**	0.77	0.64, 0.91	0.003**
**CVD**									
**Q1**	Ref	Ref		Ref	Ref		Ref	Ref	
**Q2**	0.80	0.62, 1.04	0.091	0.84	0.65, 1.08	0.167	0.84	0.65, 1.09	0.187
**Q3**	0.64	0.49, 0.84	0.001**	0.67	0.51, 0.88	0.004**	0.68	0.52, 0.90	0.006**
	**DHA**								
**ALL-cause**									
**Q1**	Ref	Ref		Ref	Ref		Ref	Ref	
**Q2**	1.09	0.95, 1.26	0.221	1.11	0.96, 1.29	0.148	1.13	0.96, 1.32	0.142
**Q3**	0.95	0.80, 1.12	0.509	0.96	0.80, 1.15	0.669	0.98	0.79, 1.20	0.832
**CVD**									
**Q1**	Ref	Ref		Ref	Ref		Ref	Ref	
**Q2**	1.08	0.86, 1.35	0.525	1.09	0.86, 1.38	0.463	1.09	0.85, 1.40	0.489
**Q3**	0.88	0.70, 1.11	0.268	0.88	0.69, 1.13	0.325	0.89	0.67, 1.19	0.437
	**EPA**								
**ALL-cause**									
**Q1**	Ref	Ref		Ref	Ref		Ref	Ref	
**Q2**	0.92	0.79, 1.06	0.242	0.94	0.82, 1.08	0.389	0.92	0.80, 1.06	0.262
**Q3**	0.84	0.71, 0.99	0.039*	0.89	0.74, 1.06	0.188	0.90	0.75, 1.08	0.252
**CVD**									
**Q1**	Ref	Ref		Ref	Ref		Ref	Ref	
**Q2**	0.85	0.66, 1.09	0.205	0.86	0.67, 1.12	0.263	0.87	0.66, 1.14	0.312
**Q3**	0.78	0.61, 1.00	0.051	0.82	0.64, 1.05	0.120	0.84	0.65, 1.10	0.202

^*^ *p* < 0.05; **p < 0.01

^a^*HR* hazard ratio, *CI *confidence interval, *CVD *CVD mortality, *ALL-cause *All-cause mortality; multivariate adjusted Cox proportional regression models of marine PUFAs intake with cardiovascular disease: Model 1: Age and race; Model 2: Model 1 + education, marital status, ratio of family income to poverty, BMI, physical activity, smoking status and alcohol consumption; Model 3: Model 2 + hypertension, diabetes, cancer, osteoarthritis, total energy intake, total fat intake, and total cholesterol intake

### Sensitivity analysis of PUFAs and CVD, all-cause mortality, CVD mortality

In a sensitivity analysis of overweight people (Supplemental Tables 1, 2, 3 and 4), follow-up of more than two years (Supplemental Tables 5, 6, 7 and 8), no extreme fat intake (Supplemental Tables 13, 14, 15 and 16), and including normolipidemic men (Supplemental Tables 21, 22, 23 and  24), the inverse association between dietary DPA intake levels and cardiovascular disease remained significant, including the risk of angina and stroke. Consistent with the main analysis, high levels of DPA intake still reduced all-cause and CVD mortality. In the overall male subgroup, the highest tertile of EPA intake was associated with a lower risk of congestive heart failure (OR = 0.60, 95%CI: 0.40, 0.89). In elderly men with dyslipidemia (Supplemental Tables 9, 10, 11 and 12), there was no significant association between intake of DPA and CVD (OR = 0.84, 95%CI: 0.63, 1.11), and other results were consistent with the main analysis. In the group of women with dyslipidemia (Supplemental Tables 17, 18, 19 and 20), dietary DPA and DHA were not associated with CVD risk. High levels of DPA and DHA intake can reduce all-cause mortality, and high levels of DPA intake can also reduce CVD mortality. Participants in the second tertile of EPA intake levels had a lower risk of stroke (OR = 0.67, 95%CI: 0.46, 1.00), compared with the lowest intake level. The second quartile DPA intake level can reduce all-cause mortality (HR = 0.85, 95%CI: 0.72, 0.99) and CVD mortality (HR = 0.73, 95%CI: 0.56, 0.97).

## Discussion

This study analyzed a nationally representative sample of 11,848 participants from the NHANES database from 2001 to 2016 to explain the association between intake of different types of marine PUFAs and cardiovascular disease and death among adult males with dyslipidemia in the United States. Dietary DPA intake in men with dyslipidemia was inversely associated with risk of CVD (mainly angina and stroke) and reduced all-cause and CVD mortality in men with dyslipidemia after adjustment for underlying influencing factors, independent of EPA and DHA intake levels. The same results were seen in the sensitivity analysis, especially for people who were overweight or obese. In the elderly population, the association between dietary DPA intake and angina was weakened, possibly because older adults may consume more PUFAs, affecting the development of CVD [16]. In women with dyslipidemia, DPA intake at the second quartile level can reduce mortality. The reason for this may be that women benefit more from PUFA supplementation [17]. In the subgroup including men with normal blood lipid levels, EPA intake was inversely associated with congestive heart failure. This may be due to the fact that the health of the body has a great influence on the absorption of food or medicine. This may be because the anti-fibrosis effect of EPA has a positive effect on the prevention of congestive heart failure, and the health status of the body has a great influence on the absorption of food or medicine [18]. However, the mechanism of action of different PUFAs on the human body is still unclear, and further research is still needed.

The benefits of EPA and DHA supplementation remain controversial. In the OMEMI trial, the higher the EPA supplementation level was used in patients with recent acute myocardial infarction, the lower the risk of major cardiovascular events and the higher the risk of new atrial fibrillation [19]. A secondary analysis of the STRENGTH test showed that Omega-3 fatty acid preparations had no effect on participants with high triglycerides and low HDL levels [20]; The EPA and DHA interventions did not reduce the levels of inflammation or markers of heart failure in patients over five years [21]. In a population study, healthy subjects receiving EPA intervention increased the oxidative sensitivity of LDL, which may lead to atherosclerosis [22]. In animal experiments, supplementation of PUFAs in maternal rats during pregnancy/lactation resulted in a significant increase in the total fat percentage of the offspring [23]. DPA is mainly found in deep-sea fish and seal oils and is relatively rare compared with EPA and DHA [24]. But recent studies have suggested that DPA may be more beneficial to the body. Previous studies showed that DPA supplement could increase the level of resolvins, protectins, and maresins in body, which was a small molecule with strong antioxidant effect, leading to reprogramming of peripheral white blood cells and significant enrichment of genes involved in immune regulation and peripheral blood cell reaction. Compared with other PUFAs, DPA may play a stronger role in regulating the body's immune capacity and cardiovascular inflammation [25–27]. In one study, supplementation with MAT9001 (a fatty acid formula rich in EPA and DPA) was found to have a better effect than ethyl eicosapentaenoate, resulting in higher levels of PUFAs in the blood circulation, lower triglycerides and high sensitivity C-reactive protein without increasing LDL [28]. In addition, another study found that dietary DPA supplementation reduced postprandial-circulation chylomicron levels and reduced the risk of cardiovascular disease, but not EPA [29].

Patients with dyslipidemia tend to have high levels of inflammation and lipid disorders, which are key risk factors for human atherosclerosis and thrombosis, as well as important risk factors for cardiovascular disease [30–32]. Previous studies have shown that DPA could adjust inflammation and blood lipid levels. An animal experiment showed that compared with the control diet, dietary DPA interfered with the down-regulation of HMG-CoA reductase in rats, resulting in lower plasma TC. And increased aortic activity in rats is mediated by inhibition of gene expression of cyclooxygenase-2 [33]. In addition, DPA inhibits arachidonic acid-stimulated platelet aggregation, interferes with the cyclooxygenase pathway and accelerates the lipoxygenase pathway, effectively reducing thrombosis and reducing the risk of cardiovascular disease due to thrombosis [34, 35].

Dyslipidemia associated with poor diet and lifestyle may be an important factor in cardiovascular disease. According to epidemiological studies, dyslipidemia is associated with increased morbidity and mortality of cardiovascular disease and can be reduced through dietary interventions, regular exercise, reaching a target weight and smoking cessation [36, 37]. Increasing the intake of PUFAs or supplementing their formulations is one possible way to ameliorate adverse factors of cardiovascular disease. In this study, intake of the highest third of DPA was associated with a reduced risk of cardiovascular disease and death in dyslipidemic male, but higher levels of EPA and DHA did not reduce the risk, consistent with the Enhanced -IT study. The mechanism by which PUFAs are associated with cardiovascular disease may require further investigation.

This study has the following limitations. First, NHANES is a cross-sectional study, and dietary data were obtained through 24-h recall, which may have a certain recall bias. Furthermore, due to the design of the study, cardiovascular disease, which we used as an endpoint, was diagnosed before the diet survey. Second, the levels of individual marine PUFAs in dietary supplements were not included in our analysis because there was no single fatty acid supplement dose in the supplements and the study did not focus on internal exposure. Third, the lifestyle and diet of the participants may change over a long period of time, and subsequent repeated measurements are required. Still, there are some strengths to our study. First, we described the association of dietary marine polyunsaturated fatty acids with different types of cardiovascular disease and all-cause and cardiovascular disease-specific mortality. Second, we used a larger sample size, which is nationally representative, and the results are more convincing. In addition, more studies focus on the role of EPA and DHA, and there are few studies on the relationship between dietary DPA and cardiovascular diseases in the population. This study may have some implications for the prevention of cardiovascular disease in dyslipidemia.

## Conclusion

In summary, higher levels of DPA but not DHA or EPA intakes were negatively associated with cardiovascular disease risk in U.S. adult male with dyslipidemia and were associated with reduced all-cause and cardiovascular mortality.

### Supplementary Information

**Additional file 1.**

## Data Availability

The publicly available data used in this study can be found here: https://www.cdc.gov/nchs/nhanes/index.htm.

## Abbreviations

BMI: Body mass index
CVD: Cardiovascular diseases
DHA: Docosahexaenoic acid
DPA: Docosapentaenoic acid
EPA: Eicosapentaenoic acid
HDL: High-density lipoprotein
HMG-CoA: Hydroxymethylglutaryl coenzyme A
HR: Hazard ratio
LDL: Low-density lipoproteins
NDI: National Death Index
NHANES: National Health and Nutrition Examination Survey
NCHS: National Center for Health Statistics
OR: Odds ratio
PUFAs: Polyunsaturated fatty acids
