# Supplementary material for: Association of marine PUFAs intakes with cardiovascular disease, all-cause mortality, and cardiovascular mortality in American adult male patients with dyslipidemia: the U.S. National Health and Nutrition Examination Survey, 2001 to 2016
Source: Nutr J. 2023 Oct 6;22:48. doi: 10.1186/s12937-023-00873-6 (PMC10557340; doi:10.1186/s12937-023-00873-6)
Supplement: Supplementary file 1 — Additional file 1. [file 12937_2023_873_MOESM1_ESM.docx]

Association of marine PUFAs intake with cardiovascular disease, all-cause mortality, and cardiovascular mortality in American adult male patients with dyslipidemia: The U.S. National Health and Nutrition Examination Survey, 2001 to 2016.

First author: Xuanfeng Tang

Supplemental Table 1

|  | **Model 1** | | | **Model 2** | | | **Model 3** | | |
| --- | --- | --- | --- | --- | --- | --- | --- | --- | --- |
|  | **OR^1^** | **95% CI^1^** | **p-value** | **OR^1^** | **95% CI^1^** | **p-value** | **OR^1^** | **95% CI^1^** | **p-value** |
| **CVD** |  |  |  |  |  |  |  |  |  |
| **Q1** | Ref | Ref |  | Ref | Ref |  | Ref | Ref |  |
| **Q2** | 0.87 | 0.68, 1.10 | 0.237 | 0.91 | 0.72, 1.15 | 0.423 | 0.91 | 0.71, 1.16 | 0.430 |
| **Q3** | 0.66 | 0.52, 0.85 | 0.001** | 0.68 | 0.52, 0.89 | 0.005** | 0.69 | 0.52, 0.92 | 0.011* |
| **CHF** |  |  |  |  |  |  |  |  |  |
| **Q1** | Ref | Ref |  | Ref | Ref |  | Ref | Ref |  |
| **Q2** | 0.90 | 0.64, 1.28 | 0.569 | 0.97 | 0.68, 1.40 | 0.881 | 0.96 | 0.64, 1.42 | 0.823 |
| **Q3** | 0.86 | 0.57, 1.31 | 0.487 | 0.91 | 0.58, 1.43 | 0.693 | 0.98 | 0.59, 1.61 | 0.933 |
| **CHD** |  |  |  |  |  |  |  |  |  |
| **Q1** | Ref | Ref |  | Ref | Ref |  | Ref | Ref |  |
| **Q2** | 0.86 | 0.64, 1.16 | 0.331 | 0.90 | 0.67, 1.21 | 0.471 | 0.93 | 0.69, 1.26 | 0.634 |
| **Q3** | 0.78 | 0.57, 1.07 | 0.121 | 0.80 | 0.57, 1.12 | 0.186 | 0.91 | 0.63, 1.32 | 0.623 |
| **Angina** |  |  |  |  |  |  |  |  |  |
| **Q1** | Ref | Ref |  | Ref | Ref |  | Ref | Ref |  |
| **Q2** | 0.71 | 0.48, 1.06 | 0.094 | 0.74 | 0.50, 1.11 | 0.149 | 0.75 | 0.51, 1.12 | 0.162 |
| **Q3** | 0.51 | 0.32, 0.80 | 0.004** | 0.52 | 0.33, 0.84 | 0.008** | 0.51 | 0.32, 0.79 | 0.003** |
| **MI** |  |  |  |  |  |  |  |  |  |
| **Q1** | Ref | Ref |  | Ref | Ref |  | Ref | Ref |  |
| **Q2** | 0.80 | 0.59, 1.07 | 0.133 | 0.84 | 0.63, 1.11 | 0.220 | 0.83 | 0.62, 1.11 | 0.218 |
| **Q3** | 0.68 | 0.48, 0.95 | 0.026* | 0.70 | 0.49, 1.00 | 0.050 | 0.69 | 0.48, 1.00 | 0.048* |
| **Stroke** |  |  |  |  |  |  |  |  |  |
| **Q1** | Ref | Ref |  | Ref | Ref |  | Ref | Ref |  |
| **Q2** | 0.86 | 0.56, 1.32 | 0.490 | 0.89 | 0.58, 1.38 | 0.613 | 0.87 | 0.57, 1.33 | 0.517 |
| **Q3** | 0.50 | 0.33, 0.76 | 0.001** | 0.51 | 0.34, 0.77 | 0.002** | 0.52 | 0.34, 0.81 | 0.005** |

Supplemental Table 2

|  | **Model 1** | | | **Model 2** | | | **Model 3** | | |
| --- | --- | --- | --- | --- | --- | --- | --- | --- | --- |
|  | **OR^1^** | **95% CI^1^** | **p-value** | **OR^1^** | **95% CI^1^** | **p-value** | **OR^1^** | **95% CI^1^** | **p-value** |
| **CVD** |  |  |  |  |  |  |  |  |  |
| **Q1** | Ref | Ref |  | Ref | Ref |  | Ref | Ref |  |
| **Q2** | 0.88 | 0.66, 1.18 | 0.381 | 0.90 | 0.67, 1.21 | 0.496 | 0.96 | 0.68, 1.33 | 0.789 |
| **Q3** | 0.85 | 0.65, 1.12 | 0.258 | 0.87 | 0.65, 1.16 | 0.350 | 0.94 | 0.67, 1.31 | 0.708 |
| **CHF** |  |  |  |  |  |  |  |  |  |
| **Q1** | Ref | Ref |  | Ref | Ref |  | Ref | Ref |  |
| **Q2** | 0.99 | 0.68, 1.44 | 0.944 | 1.03 | 0.71, 1.50 | 0.868 | 1.10 | 0.72, 1.68 | 0.642 |
| **Q3** | 1.01 | 0.66, 1.55 | 0.950 | 1.04 | 0.66, 1.62 | 0.871 | 1.16 | 0.69, 1.96 | 0.567 |
| **CHD** |  |  |  |  |  |  |  |  |  |
| **Q1** | Ref | Ref |  | Ref | Ref |  | Ref | Ref |  |
| **Q2** | 0.81 | 0.56, 1.15 | 0.235 | 0.82 | 0.57, 1.17 | 0.261 | 0.93 | 0.63, 1.38 | 0.731 |
| **Q3** | 0.84 | 0.61, 1.16 | 0.283 | 0.84 | 0.60, 1.18 | 0.307 | 1.01 | 0.69, 1.48 | 0.961 |
| **Angina** |  |  |  |  |  |  |  |  |  |
| **Q1** | Ref | Ref |  | Ref | Ref |  | Ref | Ref |  |
| **Q2** | 0.80 | 0.49, 1.29 | 0.352 | 0.83 | 0.51, 1.33 | 0.427 | 0.93 | 0.58, 1.49 | 0.754 |
| **Q3** | 0.99 | 0.62, 1.60 | 0.981 | 1.03 | 0.63, 1.69 | 0.898 | 1.17 | 0.71, 1.92 | 0.541 |
| **MI** |  |  |  |  |  |  |  |  |  |
| **Q1** | Ref | Ref |  | Ref | Ref |  | Ref | Ref |  |
| **Q2** | 1.01 | 0.77, 1.33 | 0.941 | 1.05 | 0.79, 1.40 | 0.737 | 1.02 | 0.75, 1.40 | 0.897 |
| **Q3** | 0.92 | 0.66, 1.28 | 0.611 | 0.94 | 0.66, 1.33 | 0.715 | 0.89 | 0.60, 1.33 | 0.558 |
| **Stroke** |  |  |  |  |  |  |  |  |  |
| **Q1** | Ref | Ref |  | Ref | Ref |  | Ref | Ref |  |
| **Q2** | 0.87 | 0.58, 1.29 | 0.486 | 0.91 | 0.61, 1.36 | 0.654 | 0.99 | 0.63, 1.54 | 0.949 |
| **Q3** | 0.71 | 0.49, 1.03 | 0.072 | 0.75 | 0.51, 1.09 | 0.127 | 0.84 | 0.54, 1.30 | 0.420 |

Supplemental Table 3

|  | **Model 1** | | | **Model 2** | | | **Model 3** | | |
| --- | --- | --- | --- | --- | --- | --- | --- | --- | --- |
|  | **OR^1^** | **95% CI^1^** | **p-value** | **OR^1^** | **95% CI^1^** | **p-value** | **OR^1^** | **95% CI^1^** | **p-value** |
| **CVD** |  |  |  |  |  |  |  |  |  |
| **Q1** | Ref | Ref |  | Ref | Ref |  | Ref | Ref |  |
| **Q2** | 0.97 | 0.73, 1.28 | 0.810 | 1.01 | 0.76, 1.35 | 0.932 | 1.03 | 0.76, 1.38 | 0.867 |
| **Q3** | 0.79 | 0.61, 1.04 | 0.096 | 0.86 | 0.65, 1.15 | 0.305 | 0.89 | 0.66, 1.21 | 0.466 |
| **CHF** |  |  |  |  |  |  |  |  |  |
| **Q1** | Ref | Ref |  | Ref | Ref |  | Ref | Ref |  |
| **Q2** | 1.12 | 0.79, 1.58 | 0.514 | 1.22 | 0.87, 1.72 | 0.247 | 1.26 | 0.89, 1.76 | 0.187 |
| **Q3** | 0.90 | 0.60, 1.37 | 0.627 | 0.99 | 0.64, 1.53 | 0.970 | 1.04 | 0.69, 1.58 | 0.843 |
| **CHD** |  |  |  |  |  |  |  |  |  |
| **Q1** | Ref | Ref |  | Ref | Ref |  | Ref | Ref |  |
| **Q2** | 0.86 | 0.61, 1.22 | 0.405 | 0.90 | 0.63, 1.27 | 0.534 | 0.94 | 0.66, 1.34 | 0.730 |
| **Q3** | 0.90 | 0.66, 1.21 | 0.470 | 0.95 | 0.70, 1.29 | 0.738 | 1.06 | 0.76, 1.46 | 0.744 |
| **Angina** |  |  |  |  |  |  |  |  |  |
| **Q1** | Ref | Ref |  | Ref | Ref |  | Ref | Ref |  |
| **Q2** | 0.90 | 0.61, 1.34 | 0.605 | 0.96 | 0.64, 1.45 | 0.844 | 1.00 | 0.66, 1.49 | 0.984 |
| **Q3** | 0.80 | 0.53, 1.21 | 0.297 | 0.89 | 0.58, 1.39 | 0.613 | 0.93 | 0.59, 1.48 | 0.770 |
| **MI** |  |  |  |  |  |  |  |  |  |
| **Q1** | Ref | Ref |  | Ref | Ref |  | Ref | Ref |  |
| **Q2** | 0.94 | 0.68, 1.32 | 0.728 | 1.00 | 0.72, 1.40 | 0.982 | 1.02 | 0.74, 1.40 | 0.922 |
| **Q3** | 0.69 | 0.50, 0.94 | 0.021* | 0.75 | 0.54, 1.05 | 0.092 | 0.76 | 0.54, 1.06 | 0.107 |
| **Stroke** |  |  |  |  |  |  |  |  |  |
| **Q1** | Ref | Ref |  | Ref | Ref |  | Ref | Ref |  |
| **Q2** | 0.75 | 0.52, 1.09 | 0.127 | 0.78 | 0.54, 1.12 | 0.171 | 0.79 | 0.51, 1.21 | 0.272 |
| **Q3** | 0.56 | 0.38, 0.84 | 0.005** | 0.62 | 0.41, 0.91 | 0.017* | 0.64 | 0.41, 1.02 | 0.062 |

Supplemental Table 4

|  | **Model 1** | | | **Model 2** | | | **Model 3** | | |
| --- | --- | --- | --- | --- | --- | --- | --- | --- | --- |
|  | **HR1** | **95% CI1** | **p-value** | **HR1** | **95% CI1** | **p-value** | **HR1** | **95% CI1** | **p-value** |
|  | **DPA** |  |  |  |  |  |  |  |  |
| **ALL-cause** |  |  |  |  |  |  |  |  |  |
| **Q1** | Ref | Ref |  | Ref | Ref |  | Ref | Ref |  |
| **Q2** | 0.80 | 0.65, 0.99 | 0.038* | 0.85 | 0.71, 1.03 | 0.102 | 0.86 | 0.71, 1.03 | 0.108 |
| **Q3** | 0.71 | 0.57, 0.88 | 0.002** | 0.73 | 0.58, 0.91 | 0.006** | 0.73 | 0.59, 0.91 | 0.004** |
| **CVD** |  |  |  |  |  |  |  |  |  |
| **Q1** | Ref | Ref |  | Ref | Ref |  | Ref | Ref |  |
| **Q2** | 0.85 | 0.62, 1.16 | 0.304 | 0.90 | 0.67, 1.22 | 0.515 | 0.92 | 0.68, 1.25 | 0.602 |
| **Q3** | 0.61 | 0.44, 0.85 | 0.003** | 0.63 | 0.45, 0.89 | 0.008** | 0.64 | 0.46, 0.89 | 0.009** |
|  | **DHA** |  |  |  |  |  |  |  |  |
| **ALL-cause** |  |  |  |  |  |  |  |  |  |
| **Q1** | Ref | Ref |  | Ref | Ref |  | Ref | Ref |  |
| **Q2** | 1.03 | 0.87, 1.21 | 0.764 | 1.06 | 0.89, 1.26 | 0.530 | 1.08 | 0.89, 1.31 | 0.451 |
| **Q3** | 0.94 | 0.77, 1.16 | 0.589 | 0.94 | 0.76, 1.17 | 0.596 | 0.96 | 0.76, 1.21 | 0.735 |
| **CVD** |  |  |  |  |  |  |  |  |  |
| **Q1** | Ref | Ref |  | Ref | Ref |  | Ref | Ref |  |
| **Q2** | 1.10 | 0.81, 1.50 | 0.540 | 1.15 | 0.83, 1.58 | 0.396 | 1.12 | 0.81, 1.56 | 0.487 |
| **Q3** | 0.91 | 0.68, 1.22 | 0.522 | 0.92 | 0.68, 1.24 | 0.582 | 0.89 | 0.63, 1.26 | 0.506 |
|  | **EPA** |  |  |  |  |  |  |  |  |
| **ALL-cause** |  |  |  |  |  |  |  |  |  |
| **Q1** | Ref | Ref |  | Ref | Ref |  | Ref | Ref |  |
| **Q2** | 0.92 | 0.77, 1.10 | 0.348 | 0.95 | 0.79, 1.15 | 0.615 | 0.94 | 0.78, 1.12 | 0.465 |
| **Q3** | 0.83 | 0.67, 1.03 | 0.094 | 0.85 | 0.66, 1.08 | 0.175 | 0.84 | 0.67, 1.06 | 0.152 |
| **CVD** |  |  |  |  |  |  |  |  |  |
| **Q1** | Ref | Ref |  | Ref | Ref |  | Ref | Ref |  |
| **Q2** | 0.76 | 0.55, 1.05 | 0.096 | 0.79 | 0.57, 1.10 | 0.166 | 0.79 | 0.56, 1.11 | 0.178 |
| **Q3** | 0.71 | 0.52, 0.95 | 0.023* | 0.72 | 0.53, 1.00 | 0.047* | 0.72 | 0.51, 1.00 | 0.052 |

Supplemental Table 5

|  | **Model 1** | | | **Model 2** | | | **Model 3** | | |
| --- | --- | --- | --- | --- | --- | --- | --- | --- | --- |
|  | **OR^1^** | **95% CI^1^** | **p-value** | **OR^1^** | **95% CI^1^** | **p-value** | **OR^1^** | **95% CI^1^** | **p-value** |
| **CVD** |  |  |  |  |  |  |  |  |  |
| **Q1** | Ref | Ref |  | Ref | Ref |  | Ref | Ref |  |
| **Q2** | 0.87 | 0.70, 1.07 | 0.186 | 0.88 | 0.72, 1.09 | 0.246 | 0.86 | 0.69, 1.07 | 0.176 |
| **Q3** | 0.70 | 0.55, 0.88 | 0.003** | 0.71 | 0.56, 0.91 | 0.008** | 0.70 | 0.54, 0.91 | 0.008** |
| **CHF** |  |  |  |  |  |  |  |  |  |
| **Q1** | Ref | Ref |  | Ref | Ref |  | Ref | Ref |  |
| **Q2** | 0.87 | 0.63, 1.20 | 0.402 | 0.91 | 0.65, 1.27 | 0.570 | 0.86 | 0.61, 1.23 | 0.418 |
| **Q3** | 0.83 | 0.57, 1.19 | 0.303 | 0.86 | 0.59, 1.26 | 0.438 | 0.82 | 0.54, 1.24 | 0.344 |
| **CHD** |  |  |  |  |  |  |  |  |  |
| **Q1** | Ref | Ref |  | Ref | Ref |  | Ref | Ref |  |
| **Q2** | 0.87 | 0.66, 1.13 | 0.295 | 0.87 | 0.67, 1.13 | 0.301 | 0.89 | 0.68, 1.16 | 0.373 |
| **Q3** | 0.76 | 0.57, 1.02 | 0.065 | 0.77 | 0.57, 1.04 | 0.087 | 0.84 | 0.61, 1.17 | 0.312 |
| **Angina** |  |  |  |  |  |  |  |  |  |
| **Q1** | Ref | Ref |  | Ref | Ref |  | Ref | Ref |  |
| **Q2** | 0.73 | 0.52, 1.04 | 0.078 | 0.74 | 0.52, 1.06 | 0.100 | 0.73 | 0.51, 1.03 | 0.076 |
| **Q3** | 0.55 | 0.38, 0.80 | 0.002** | 0.56 | 0.39, 0.83 | 0.004** | 0.55 | 0.38, 0.79 | 0.002** |
| **MI** |  |  |  |  |  |  |  |  |  |
| **Q1** | Ref | Ref |  | Ref | Ref |  | Ref | Ref |  |
| **Q2** | 0.92 | 0.70, 1.21 | 0.563 | 0.95 | 0.73, 1.25 | 0.721 | 0.93 | 0.71, 1.23 | 0.620 |
| **Q3** | 0.77 | 0.58, 1.02 | 0.069 | 0.80 | 0.59, 1.08 | 0.138 | 0.76 | 0.56, 1.04 | 0.091 |
| **Stroke** |  |  |  |  |  |  |  |  |  |
| **Q1** | Ref | Ref |  | Ref | Ref |  | Ref | Ref |  |
| **Q2** | 0.87 | 0.61, 1.23 | 0.416 | 0.88 | 0.62, 1.25 | 0.468 | 0.84 | 0.59, 1.20 | 0.337 |
| **Q3** | 0.59 | 0.41, 0.83 | 0.003** | 0.60 | 0.42, 0.85 | 0.005** | 0.61 | 0.42, 0.88 | 0.010* |

Supplemental Table 6

|  | **Model 1** | | | **Model 2** | | | **Model 3** | | |
| --- | --- | --- | --- | --- | --- | --- | --- | --- | --- |
|  | **OR^1^** | **95% CI^1^** | **p-value** | **OR^1^** | **95% CI^1^** | **p-value** | **OR^1^** | **95% CI^1^** | **p-value** |
| **CVD** |  |  |  |  |  |  |  |  |  |
| **Q1** | Ref | Ref |  | Ref | Ref |  | Ref | Ref |  |
| **Q2** | 0.90 | 0.73, 1.11 | 0.300 | 0.90 | 0.73, 1.13 | 0.371 | 0.96 | 0.74, 1.24 | 0.750 |
| **Q3** | 0.88 | 0.72, 1.08 | 0.207 | 0.90 | 0.72, 1.13 | 0.369 | 0.97 | 0.75, 1.26 | 0.833 |
| **CHF** |  |  |  |  |  |  |  |  |  |
| **Q1** | Ref | Ref |  | Ref | Ref |  | Ref | Ref |  |
| **Q2** | 1.00 | 0.72, 1.38 | 0.996 | 1.03 | 0.75, 1.42 | 0.863 | 1.03 | 0.72, 1.49 | 0.869 |
| **Q3** | 0.94 | 0.66, 1.34 | 0.734 | 0.97 | 0.67, 1.40 | 0.863 | 0.96 | 0.62, 1.50 | 0.862 |
| **CHD** |  |  |  |  |  |  |  |  |  |
| **Q1** | Ref | Ref |  | Ref | Ref |  | Ref | Ref |  |
| **Q2** | 0.78 | 0.59, 1.03 | 0.081 | 0.78 | 0.59, 1.03 | 0.079 | 0.89 | 0.65, 1.22 | 0.448 |
| **Q3** | 0.87 | 0.67, 1.13 | 0.303 | 0.88 | 0.68, 1.15 | 0.357 | 1.05 | 0.77, 1.43 | 0.762 |
| **Angina** |  |  |  |  |  |  |  |  |  |
| **Q1** | Ref | Ref |  | Ref | Ref |  | Ref | Ref |  |
| **Q2** | 0.78 | 0.56, 1.10 | 0.154 | 0.80 | 0.57, 1.12 | 0.188 | 0.89 | 0.61, 1.31 | 0.560 |
| **Q3** | 0.99 | 0.68, 1.46 | 0.978 | 1.03 | 0.69, 1.54 | 0.874 | 1.17 | 0.77, 1.78 | 0.467 |
| **MI** |  |  |  |  |  |  |  |  |  |
| **Q1** | Ref | Ref |  | Ref | Ref |  | Ref | Ref |  |
| **Q2** | 1.03 | 0.82, 1.30 | 0.776 | 1.05 | 0.83, 1.33 | 0.683 | 1.03 | 0.78, 1.36 | 0.836 |
| **Q3** | 1.01 | 0.78, 1.31 | 0.954 | 1.04 | 0.78, 1.38 | 0.803 | 0.99 | 0.72, 1.36 | 0.955 |
| **Stroke** |  |  |  |  |  |  |  |  |  |
| **Q1** | Ref | Ref |  | Ref | Ref |  | Ref | Ref |  |
| **Q2** | 0.88 | 0.64, 1.21 | 0.431 | 0.90 | 0.64, 1.25 | 0.523 | 0.93 | 0.63, 1.36 | 0.702 |
| **Q3** | 0.69 | 0.49, 0.98 | 0.036* | 0.72 | 0.51, 1.01 | 0.059 | 0.76 | 0.50, 1.15 | 0.199 |

Supplemental Table 7

|  | **Model 1** | | | **Model 2** | | | **Model 3** | | |
| --- | --- | --- | --- | --- | --- | --- | --- | --- | --- |
|  | **OR^1^** | **95% CI^1^** | **p-value** | **OR^1^** | **95% CI^1^** | **p-value** | **OR^1^** | **95% CI^1^** | **p-value** |
| **CVD** |  |  |  |  |  |  |  |  |  |
| **Q1** | Ref | Ref |  | Ref | Ref |  | Ref | Ref |  |
| **Q2** | 0.96 | 0.78, 1.19 | 0.728 | 0.99 | 0.80, 1.23 | 0.962 | 1.00 | 0.80, 1.26 | 0.986 |
| **Q3** | 0.83 | 0.67, 1.04 | 0.103 | 0.91 | 0.72, 1.15 | 0.445 | 0.95 | 0.74, 1.21 | 0.672 |
| **CHF** |  |  |  |  |  |  |  |  |  |
| **Q1** | Ref | Ref |  | Ref | Ref |  | Ref | Ref |  |
| **Q2** | 1.07 | 0.80, 1.43 | 0.638 | 1.13 | 0.84, 1.52 | 0.420 | 1.11 | 0.82, 1.50 | 0.498 |
| **Q3** | 0.84 | 0.59, 1.19 | 0.314 | 0.93 | 0.65, 1.34 | 0.712 | 0.92 | 0.65, 1.32 | 0.656 |
| **CHD** |  |  |  |  |  |  |  |  |  |
| **Q1** | Ref | Ref |  | Ref | Ref |  | Ref | Ref |  |
| **Q2** | 0.86 | 0.66, 1.13 | 0.280 | 0.87 | 0.67, 1.14 | 0.319 | 0.92 | 0.69, 1.22 | 0.556 |
| **Q3** | 0.97 | 0.75, 1.24 | 0.782 | 1.02 | 0.79, 1.33 | 0.863 | 1.14 | 0.87, 1.51 | 0.329 |
| **Angina** |  |  |  |  |  |  |  |  |  |
| **Q1** | Ref | Ref |  | Ref | Ref |  | Ref | Ref |  |
| **Q2** | 1.01 | 0.71, 1.43 | 0.958 | 1.05 | 0.73, 1.52 | 0.788 | 1.08 | 0.75, 1.58 | 0.670 |
| **Q3** | 0.93 | 0.65, 1.32 | 0.671 | 1.04 | 0.72, 1.49 | 0.851 | 1.11 | 0.75, 1.64 | 0.611 |
| **MI** |  |  |  |  |  |  |  |  |  |
| **Q1** | Ref | Ref |  | Ref | Ref |  | Ref | Ref |  |
| **Q2** | 1.00 | 0.77, 1.30 | 0.976 | 1.04 | 0.80, 1.36 | 0.741 | 1.05 | 0.81, 1.36 | 0.723 |
| **Q3** | 0.81 | 0.62, 1.06 | 0.127 | 0.89 | 0.67, 1.18 | 0.421 | 0.89 | 0.67, 1.19 | 0.432 |
| **Stroke** |  |  |  |  |  |  |  |  |  |
| **Q1** | Ref | Ref |  | Ref | Ref |  | Ref | Ref |  |
| **Q2** | 0.75 | 0.54, 1.05 | 0.090 | 0.76 | 0.55, 1.07 | 0.111 | 0.77 | 0.53, 1.12 | 0.169 |
| **Q3** | 0.61 | 0.43, 0.86 | 0.005** | 0.66 | 0.47, 0.93 | 0.018* | 0.70 | 0.48, 1.01 | 0.058 |

Supplemental Table 8

|  | **Model 1** | | | **Model 2** | | | **Model 3** | | |
| --- | --- | --- | --- | --- | --- | --- | --- | --- | --- |
|  | **HR1** | **95% CI1** | **p-value** | **HR1** | **95% CI1** | **p-value** | **HR1** | **95% CI1** | **p-value** |
|  | **DPA** |  |  |  |  |  |  |  |  |
| **ALL-cause** |  |  |  |  |  |  |  |  |  |
| **Q1** | Ref | Ref |  | Ref | Ref |  | Ref | Ref |  |
| **Q2** | 0.90 | 0.75, 1.07 | 0.221 | 0.95 | 0.80, 1.12 | 0.509 | 0.93 | 0.79, 1.10 | 0.414 |
| **Q3** | 0.74 | 0.61, 0.89 | 0.001** | 0.77 | 0.63, 0.94 | 0.011* | 0.77 | 0.63, 0.94 | 0.010* |
| **CVD** |  |  |  |  |  |  |  |  |  |
| **Q1** | Ref | Ref |  | Ref | Ref |  | Ref | Ref |  |
| **Q2** | 0.85 | 0.65, 1.12 | 0.255 | 0.90 | 0.68, 1.18 | 0.431 | 0.90 | 0.69, 1.18 | 0.441 |
| **Q3** | 0.65 | 0.49, 0.85 | 0.002** | 0.67 | 0.50, 0.90 | 0.008** | 0.68 | 0.51, 0.91 | 0.008** |
|  | **DHA** |  |  |  |  |  |  |  |  |
| **ALL-cause** |  |  |  |  |  |  |  |  |  |
| **Q1** | Ref | Ref |  | Ref | Ref |  | Ref | Ref |  |
| **Q2** | 1.10 | 0.96, 1.27 | 0.164 | 1.12 | 0.97, 1.30 | 0.111 | 1.14 | 0.98, 1.33 | 0.095 |
| **Q3** | 0.99 | 0.83, 1.17 | 0.885 | 1.00 | 0.83, 1.22 | 0.960 | 1.03 | 0.83, 1.28 | 0.798 |
| **CVD** |  |  |  |  |  |  |  |  |  |
| **Q1** | Ref | Ref |  | Ref | Ref |  | Ref | Ref |  |
| **Q2** | 1.01 | 0.79, 1.29 | 0.933 | 1.03 | 0.79, 1.33 | 0.843 | 1.02 | 0.78, 1.34 | 0.876 |
| **Q3** | 0.89 | 0.69, 1.16 | 0.393 | 0.90 | 0.69, 1.18 | 0.449 | 0.90 | 0.66, 1.23 | 0.503 |
|  | **EPA** |  |  |  |  |  |  |  |  |
| **ALL-cause** |  |  |  |  |  |  |  |  |  |
| **Q1** | Ref | Ref |  | Ref | Ref |  | Ref | Ref |  |
| **Q2** | 0.96 | 0.83, 1.11 | 0.583 | 0.98 | 0.85, 1.13 | 0.795 | 0.95 | 0.82, 1.10 | 0.529 |
| **Q3** | 0.85 | 0.71, 1.03 | 0.092 | 0.90 | 0.73, 1.09 | 0.276 | 0.90 | 0.74, 1.10 | 0.300 |
| **CVD** |  |  |  |  |  |  |  |  |  |
| **Q1** | Ref | Ref |  | Ref | Ref |  | Ref | Ref |  |
| **Q2** | 0.84 | 0.64, 1.09 | 0.188 | 0.85 | 0.65, 1.11 | 0.235 | 0.84 | 0.63, 1.11 | 0.218 |
| **Q3** | 0.79 | 0.60, 1.03 | 0.082 | 0.82 | 0.62, 1.08 | 0.159 | 0.82 | 0.62, 1.09 | 0.177 |

Supplemental Table 9

|  | **Model 1** | | | **Model 2** | | | **Model 3** | | |
| --- | --- | --- | --- | --- | --- | --- | --- | --- | --- |
|  | **OR^1^** | **95% CI^1^** | **p-value** | **OR^1^** | **95% CI^1^** | **p-value** | **OR^1^** | **95% CI^1^** | **p-value** |
| **CVD** |  |  |  |  |  |  |  |  |  |
| **Q1** | Ref | Ref |  | Ref | Ref |  | Ref | Ref |  |
| **Q2** | 0.85 | 0.70, 1.03 | 0.100 | 0.86 | 0.71, 1.05 | 0.136 | 0.85 | 0.70, 1.04 | 0.109 |
| **Q3** | 0.71 | 0.57, 0.89 | 0.003** | 0.73 | 0.58, 0.93 | 0.011* | 0.74 | 0.57, 0.95 | 0.017* |
| **CHF** |  |  |  |  |  |  |  |  |  |
| **Q1** | Ref | Ref |  | Ref | Ref |  | Ref | Ref |  |
| **Q2** | 0.90 | 0.68, 1.21 | 0.495 | 0.94 | 0.70, 1.28 | 0.712 | 0.91 | 0.66, 1.26 | 0.579 |
| **Q3** | 0.91 | 0.65, 1.27 | 0.564 | 0.95 | 0.67, 1.36 | 0.787 | 0.94 | 0.64, 1.38 | 0.743 |
| **CHD** |  |  |  |  |  |  |  |  |  |
| **Q1** | Ref | Ref |  | Ref | Ref |  | Ref | Ref |  |
| **Q2** | 0.81 | 0.64, 1.03 | 0.091 | 0.81 | 0.64, 1.03 | 0.090 | 0.83 | 0.64, 1.06 | 0.132 |
| **Q3** | 0.77 | 0.59, 1.00 | 0.052 | 0.78 | 0.59, 1.03 | 0.081 | 0.85 | 0.62, 1.16 | 0.290 |
| **Angina** |  |  |  |  |  |  |  |  |  |
| **Q1** | Ref | Ref |  | Ref | Ref |  | Ref | Ref |  |
| **Q2** | 0.71 | 0.51, 0.99 | 0.046* | 0.71 | 0.51, 1.00 | 0.053 | 0.70 | 0.49, 0.98 | 0.039* |
| **Q3** | 0.57 | 0.39, 0.82 | 0.003** | 0.58 | 0.40, 0.85 | 0.005** | 0.55 | 0.37, 0.80 | 0.002** |
| **MI** |  |  |  |  |  |  |  |  |  |
| **Q1** | Ref | Ref |  | Ref | Ref |  | Ref | Ref |  |
| **Q2** | 0.88 | 0.68, 1.14 | 0.337 | 0.90 | 0.70, 1.17 | 0.438 | 0.90 | 0.69, 1.17 | 0.410 |
| **Q3** | 0.80 | 0.60, 1.06 | 0.124 | 0.83 | 0.61, 1.13 | 0.234 | 0.83 | 0.60, 1.13 | 0.233 |
| **Stroke** |  |  |  |  |  |  |  |  |  |
| **Q1** | Ref | Ref |  | Ref | Ref |  | Ref | Ref |  |
| **Q2** | 0.83 | 0.60, 1.15 | 0.264 | 0.85 | 0.61, 1.18 | 0.320 | 0.82 | 0.59, 1.15 | 0.251 |
| **Q3** | 0.60 | 0.43, 0.83 | 0.003** | 0.61 | 0.44, 0.86 | 0.005** | 0.64 | 0.43, 0.94 | 0.022* |

Supplemental Table 10

|  | **Model 1** | | | **Model 2** | | | **Model 3** | | |
| --- | --- | --- | --- | --- | --- | --- | --- | --- | --- |
|  | **OR^1^** | **95% CI^1^** | **p-value** | **OR^1^** | **95% CI^1^** | **p-value** | **OR^1^** | **95% CI^1^** | **p-value** |
| **CVD** |  |  |  |  |  |  |  |  |  |
| **Q1** | Ref | Ref |  | Ref | Ref |  | Ref | Ref |  |
| **Q2** | 0.89 | 0.73, 1.09 | 0.262 | 0.90 | 0.73, 1.12 | 0.334 | 0.95 | 0.74, 1.22 | 0.665 |
| **Q3** | 0.88 | 0.72, 1.07 | 0.203 | 0.91 | 0.73, 1.13 | 0.375 | 0.98 | 0.76, 1.26 | 0.867 |
| **CHF** |  |  |  |  |  |  |  |  |  |
| **Q1** | Ref | Ref |  | Ref | Ref |  | Ref | Ref |  |
| **Q2** | 1.06 | 0.77, 1.45 | 0.725 | 1.09 | 0.80, 1.48 | 0.594 | 1.09 | 0.77, 1.56 | 0.619 |
| **Q3** | 1.00 | 0.71, 1.41 | 0.997 | 1.04 | 0.72, 1.48 | 0.849 | 1.06 | 0.69, 1.62 | 0.791 |
| **CHD** |  |  |  |  |  |  |  |  |  |
| **Q1** | Ref | Ref |  | Ref | Ref |  | Ref | Ref |  |
| **Q2** | 0.80 | 0.61, 1.04 | 0.090 | 0.80 | 0.61, 1.04 | 0.091 | 0.88 | 0.65, 1.19 | 0.393 |
| **Q3** | 0.86 | 0.68, 1.11 | 0.245 | 0.88 | 0.68, 1.13 | 0.304 | 1.00 | 0.74, 1.36 | 0.979 |
| **Angina** |  |  |  |  |  |  |  |  |  |
| **Q1** | Ref | Ref |  | Ref | Ref |  | Ref | Ref |  |
| **Q2** | 0.81 | 0.58, 1.12 | 0.200 | 0.82 | 0.59, 1.14 | 0.239 | 0.87 | 0.60, 1.25 | 0.440 |
| **Q3** | 0.99 | 0.69, 1.43 | 0.977 | 1.03 | 0.70, 1.51 | 0.871 | 1.08 | 0.73, 1.62 | 0.691 |
| **MI** |  |  |  |  |  |  |  |  |  |
| **Q1** | Ref | Ref |  | Ref | Ref |  | Ref | Ref |  |
| **Q2** | 0.99 | 0.80, 1.24 | 0.963 | 1.01 | 0.80, 1.27 | 0.947 | 0.98 | 0.75, 1.29 | 0.896 |
| **Q3** | 0.98 | 0.77, 1.25 | 0.880 | 1.01 | 0.77, 1.32 | 0.944 | 0.97 | 0.71, 1.32 | 0.847 |
| **Stroke** |  |  |  |  |  |  |  |  |  |
| **Q1** | Ref | Ref |  | Ref | Ref |  | Ref | Ref |  |
| **Q2** | 0.86 | 0.63, 1.18 | 0.356 | 0.88 | 0.63, 1.22 | 0.436 | 0.91 | 0.63, 1.32 | 0.615 |
| **Q3** | 0.71 | 0.51, 0.99 | 0.042* | 0.74 | 0.53, 1.03 | 0.072 | 0.79 | 0.53, 1.19 | 0.255 |

Supplemental Table 11

|  | **Model 1** | | | **Model 2** | | | **Model 3** | | |
| --- | --- | --- | --- | --- | --- | --- | --- | --- | --- |
|  | **OR^1^** | **95% CI^1^** | **p-value** | **OR^1^** | **95% CI^1^** | **p-value** | **OR^1^** | **95% CI^1^** | **p-value** |
| **CVD** |  |  |  |  |  |  |  |  |  |
| **Q1** | Ref | Ref |  | Ref | Ref |  | Ref | Ref |  |
| **Q2** | 1.02 | 0.82, 1.25 | 0.886 | 1.06 | 0.85, 1.31 | 0.623 | 1.07 | 0.85, 1.34 | 0.562 |
| **Q3** | 0.83 | 0.68, 1.02 | 0.083 | 0.92 | 0.74, 1.15 | 0.472 | 0.98 | 0.77, 1.24 | 0.856 |
| **CHF** |  |  |  |  |  |  |  |  |  |
| **Q1** | Ref | Ref |  | Ref | Ref |  | Ref | Ref |  |
| **Q2** | 1.15 | 0.87, 1.53 | 0.318 | 1.23 | 0.92, 1.63 | 0.161 | 1.22 | 0.91, 1.62 | 0.180 |
| **Q3** | 0.89 | 0.63, 1.24 | 0.470 | 1.01 | 0.71, 1.43 | 0.975 | 1.03 | 0.74, 1.45 | 0.844 |
| **CHD** |  |  |  |  |  |  |  |  |  |
| **Q1** | Ref | Ref |  | Ref | Ref |  | Ref | Ref |  |
| **Q2** | 0.92 | 0.71, 1.20 | 0.547 | 0.94 | 0.72, 1.23 | 0.636 | 0.98 | 0.74, 1.29 | 0.881 |
| **Q3** | 0.95 | 0.75, 1.21 | 0.682 | 1.02 | 0.79, 1.31 | 0.888 | 1.14 | 0.87, 1.49 | 0.351 |
| **Angina** |  |  |  |  |  |  |  |  |  |
| **Q1** | Ref | Ref |  | Ref | Ref |  | Ref | Ref |  |
| **Q2** | 1.00 | 0.71, 1.39 | 0.978 | 1.04 | 0.73, 1.48 | 0.835 | 1.05 | 0.74, 1.51 | 0.772 |
| **Q3** | 0.88 | 0.63, 1.22 | 0.431 | 0.98 | 0.70, 1.39 | 0.929 | 1.03 | 0.71, 1.50 | 0.877 |
| **MI** |  |  |  |  |  |  |  |  |  |
| **Q1** | Ref | Ref |  | Ref | Ref |  | Ref | Ref |  |
| **Q2** | 1.06 | 0.83, 1.34 | 0.648 | 1.11 | 0.87, 1.40 | 0.400 | 1.12 | 0.89, 1.42 | 0.314 |
| **Q3** | 0.81 | 0.63, 1.04 | 0.093 | 0.90 | 0.69, 1.17 | 0.412 | 0.92 | 0.70, 1.22 | 0.571 |
| **Stroke** |  |  |  |  |  |  |  |  |  |
| **Q1** | Ref | Ref |  | Ref | Ref |  | Ref | Ref |  |
| **Q2** | 0.76 | 0.55, 1.05 | 0.091 | 0.79 | 0.57, 1.08 | 0.138 | 0.79 | 0.55, 1.14 | 0.208 |
| **Q3** | 0.60 | 0.43, 0.84 | 0.003** | 0.66 | 0.47, 0.93 | 0.016* | 0.71 | 0.48, 1.04 | 0.076 |

Supplemental Table 12

|  | **Model 1** | | | **Model 2** | | | **Model 3** | | |
| --- | --- | --- | --- | --- | --- | --- | --- | --- | --- |
|  | **HR1** | **95% CI1** | **p-value** | **HR1** | **95% CI1** | **p-value** | **HR1** | **95% CI1** | **p-value** |
|  | **DPA** |  |  |  |  |  |  |  |  |
| **ALL-cause** |  |  |  |  |  |  |  |  |  |
| **Q1** | Ref | Ref |  | Ref | Ref |  | Ref | Ref |  |
| **Q2** | 0.81 | 0.69, 0.97 | 0.018* | 0.85 | 0.72, 1.00 | 0.044* | 0.85 | 0.72, 1.00 | 0.044* |
| **Q3** | 0.75 | 0.63, 0.88 | <0.001** | 0.77 | 0.65, 0.93 | 0.005** | 0.79 | 0.66, 0.94 | 0.007** |
| **CVD** |  |  |  |  |  |  |  |  |  |
| **Q1** | Ref | Ref |  | Ref | Ref |  | Ref | Ref |  |
| **Q2** | 0.78 | 0.60, 1.00 | 0.049* | 0.80 | 0.62, 1.03 | 0.083 | 0.81 | 0.63, 1.04 | 0.101 |
| **Q3** | 0.66 | 0.50, 0.86 | 0.002** | 0.68 | 0.52, 0.89 | 0.005** | 0.68 | 0.52, 0.90 | 0.007** |
|  | **DHA** |  |  |  |  |  |  |  |  |
| **ALL-cause** |  |  |  |  |  |  |  |  |  |
| **Q1** | Ref | Ref |  | Ref | Ref |  | Ref | Ref |  |
| **Q2** | 1.08 | 0.94, 1.25 | 0.267 | 1.11 | 0.96, 1.28 | 0.167 | 1.12 | 0.96, 1.31 | 0.143 |
| **Q3** | 0.95 | 0.80, 1.12 | 0.514 | 0.96 | 0.80, 1.15 | 0.645 | 0.98 | 0.80, 1.21 | 0.873 |
| **CVD** |  |  |  |  |  |  |  |  |  |
| **Q1** | Ref | Ref |  | Ref | Ref |  | Ref | Ref |  |
| **Q2** | 1.08 | 0.86, 1.35 | 0.534 | 1.09 | 0.86, 1.39 | 0.459 | 1.08 | 0.84, 1.40 | 0.550 |
| **Q3** | 0.89 | 0.70, 1.12 | 0.319 | 0.89 | 0.70, 1.14 | 0.365 | 0.89 | 0.65, 1.20 | 0.429 |
|  | **EPA** |  |  |  |  |  |  |  |  |
| **ALL-cause** |  |  |  |  |  |  |  |  |  |
| **Q1** | Ref | Ref |  | Ref | Ref |  | Ref | Ref |  |
| **Q2** | 0.92 | 0.80, 1.06 | 0.236 | 0.94 | 0.82, 1.07 | 0.349 | 0.92 | 0.80, 1.06 | 0.260 |
| **Q3** | 0.83 | 0.70, 0.98 | 0.033* | 0.87 | 0.72, 1.05 | 0.148 | 0.89 | 0.74, 1.08 | 0.239 |
| **CVD** |  |  |  |  |  |  |  |  |  |
| **Q1** | Ref | Ref |  | Ref | Ref |  | Ref | Ref |  |
| **Q2** | 0.85 | 0.66, 1.10 | 0.216 | 0.86 | 0.67, 1.12 | 0.271 | 0.86 | 0.65, 1.13 | 0.280 |
| **Q3** | 0.77 | 0.60, 0.99 | 0.041* | 0.81 | 0.63, 1.04 | 0.094 | 0.82 | 0.63, 1.08 | 0.155 |

Supplemental Table 13

|  | **Model 1** | | | **Model 2** | | | **Model 3** | | |
| --- | --- | --- | --- | --- | --- | --- | --- | --- | --- |
|  | **OR^1^** | **95% CI^1^** | **p-value** | **OR^1^** | **95% CI^1^** | **p-value** | **OR^1^** | **95% CI^1^** | **p-value** |
| **CVD** |  |  |  |  |  |  |  |  |  |
| **Q1** | Ref | Ref |  | Ref | Ref |  | Ref | Ref |  |
| **Q2** | 1.09 | 0.87, 1.38 | 0.451 | 1.15 | 0.91, 1.44 | 0.242 | 1.13 | 0.89, 1.43 | 0.321 |
| **Q3** | 0.80 | 0.62, 1.04 | 0.096 | 0.84 | 0.64, 1.10 | 0.192 | 0.84 | 0.63, 1.11 | 0.216 |
| **CHF** |  |  |  |  |  |  |  |  |  |
| **Q1** | Ref | Ref |  | Ref | Ref |  | Ref | Ref |  |
| **Q2** | 1.23 | 0.86, 1.76 | 0.261 | 1.31 | 0.90, 1.90 | 0.159 | 1.25 | 0.85, 1.85 | 0.252 |
| **Q3** | 1.10 | 0.75, 1.60 | 0.630 | 1.17 | 0.79, 1.72 | 0.435 | 1.05 | 0.68, 1.60 | 0.835 |
| **CHD** |  |  |  |  |  |  |  |  |  |
| **Q1** | Ref | Ref |  | Ref | Ref |  | Ref | Ref |  |
| **Q2** | 1.03 | 0.75, 1.40 | 0.863 | 1.03 | 0.76, 1.41 | 0.837 | 1.02 | 0.75, 1.38 | 0.905 |
| **Q3** | 0.88 | 0.64, 1.22 | 0.442 | 0.90 | 0.65, 1.24 | 0.503 | 0.93 | 0.67, 1.30 | 0.676 |
| **Angina** |  |  |  |  |  |  |  |  |  |
| **Q1** | Ref | Ref |  | Ref | Ref |  | Ref | Ref |  |
| **Q2** | 0.96 | 0.65, 1.42 | 0.830 | 0.99 | 0.67, 1.47 | 0.959 | 0.98 | 0.66, 1.44 | 0.908 |
| **Q3** | 0.74 | 0.48, 1.13 | 0.163 | 0.77 | 0.50, 1.20 | 0.253 | 0.77 | 0.50, 1.19 | 0.241 |
| **MI** |  |  |  |  |  |  |  |  |  |
| **Q1** | Ref | Ref |  | Ref | Ref |  | Ref | Ref |  |
| **Q2** | 0.94 | 0.69, 1.28 | 0.676 | 0.96 | 0.69, 1.34 | 0.830 | 0.95 | 0.68, 1.32 | 0.754 |
| **Q3** | 0.83 | 0.60, 1.15 | 0.262 | 0.86 | 0.60, 1.23 | 0.403 | 0.84 | 0.58, 1.24 | 0.379 |
| **Stroke** |  |  |  |  |  |  |  |  |  |
| **Q1** | Ref | Ref |  | Ref | Ref |  | Ref | Ref |  |
| **Q2** | 0.85 | 0.62, 1.17 | 0.318 | 0.91 | 0.66, 1.25 | 0.545 | 0.87 | 0.62, 1.22 | 0.417 |
| **Q3** | 0.63 | 0.45, 0.87 | 0.006** | 0.67 | 0.48, 0.94 | 0.020* | 0.69 | 0.46, 1.01 | 0.059 |

Supplemental Table 14

|  | **Model 1** | | | **Model 2** | | | **Model 3** | | |
| --- | --- | --- | --- | --- | --- | --- | --- | --- | --- |
|  | **OR^1^** | **95% CI^1^** | **p-value** | **OR^1^** | **95% CI^1^** | **p-value** | **OR^1^** | **95% CI^1^** | **p-value** |
| **CVD** |  |  |  |  |  |  |  |  |  |
| **Q1** | Ref | Ref |  | Ref | Ref |  | Ref | Ref |  |
| **Q2** | 0.86 | 0.67, 1.09 | 0.207 | 0.86 | 0.67, 1.09 | 0.214 | 0.91 | 0.69, 1.19 | 0.478 |
| **Q3** | 0.82 | 0.65, 1.03 | 0.088 | 0.83 | 0.65, 1.05 | 0.117 | 0.88 | 0.67, 1.17 | 0.379 |
| **CHF** |  |  |  |  |  |  |  |  |  |
| **Q1** | Ref | Ref |  | Ref | Ref |  | Ref | Ref |  |
| **Q2** | 1.24 | 0.84, 1.83 | 0.284 | 1.27 | 0.87, 1.85 | 0.218 | 1.18 | 0.77, 1.80 | 0.450 |
| **Q3** | 1.01 | 0.69, 1.47 | 0.965 | 1.03 | 0.69, 1.53 | 0.899 | 0.91 | 0.57, 1.46 | 0.700 |
| **CHD** |  |  |  |  |  |  |  |  |  |
| **Q1** | Ref | Ref |  | Ref | Ref |  | Ref | Ref |  |
| **Q2** | 0.79 | 0.58, 1.07 | 0.131 | 0.79 | 0.58, 1.07 | 0.127 | 0.86 | 0.61, 1.21 | 0.384 |
| **Q3** | 0.83 | 0.63, 1.10 | 0.189 | 0.84 | 0.63, 1.11 | 0.213 | 0.93 | 0.68, 1.29 | 0.677 |
| **Angina** |  |  |  |  |  |  |  |  |  |
| **Q1** | Ref | Ref |  | Ref | Ref |  | Ref | Ref |  |
| **Q2** | 0.91 | 0.60, 1.40 | 0.679 | 0.93 | 0.61, 1.43 | 0.738 | 1.06 | 0.67, 1.67 | 0.798 |
| **Q3** | 0.98 | 0.66, 1.45 | 0.919 | 1.01 | 0.67, 1.51 | 0.963 | 1.15 | 0.76, 1.76 | 0.506 |
| **MI** |  |  |  |  |  |  |  |  |  |
| **Q1** | Ref | Ref |  | Ref | Ref |  | Ref | Ref |  |
| **Q2** | 1.04 | 0.80, 1.36 | 0.778 | 1.05 | 0.80, 1.37 | 0.735 | 1.01 | 0.75, 1.37 | 0.932 |
| **Q3** | 0.87 | 0.67, 1.14 | 0.319 | 0.88 | 0.66, 1.18 | 0.395 | 0.84 | 0.60, 1.19 | 0.321 |
| **Stroke** |  |  |  |  |  |  |  |  |  |
| **Q1** | Ref | Ref |  | Ref | Ref |  | Ref | Ref |  |
| **Q2** | 0.73 | 0.52, 1.04 | 0.079 | 0.75 | 0.53, 1.06 | 0.097 | 0.76 | 0.51, 1.16 | 0.203 |
| **Q3** | 0.76 | 0.52, 1.12 | 0.159 | 0.77 | 0.53, 1.14 | 0.188 | 0.83 | 0.52, 1.33 | 0.439 |

Supplemental Table 15

|  | **Model 1** | | | **Model 2** | | | **Model 3** | | |
| --- | --- | --- | --- | --- | --- | --- | --- | --- | --- |
|  | **OR^1^** | **95% CI^1^** | **p-value** | **OR^1^** | **95% CI^1^** | **p-value** | **OR^1^** | **95% CI^1^** | **p-value** |
| **CVD** |  |  |  |  |  |  |  |  |  |
| **Q1** | Ref | Ref |  | Ref | Ref |  | Ref | Ref |  |
| **Q2** | 1.03 | 0.81, 1.31 | 0.808 | 1.07 | 0.84, 1.36 | 0.584 | 1.13 | 0.88, 1.47 | 0.337 |
| **Q3** | 0.86 | 0.68, 1.09 | 0.207 | 0.91 | 0.72, 1.16 | 0.463 | 0.96 | 0.75, 1.23 | 0.770 |
| **CHF** |  |  |  |  |  |  |  |  |  |
| **Q1** | Ref | Ref |  | Ref | Ref |  | Ref | Ref |  |
| **Q2** | 1.24 | 0.86, 1.80 | 0.247 | 1.32 | 0.91, 1.91 | 0.148 | 1.27 | 0.87, 1.85 | 0.206 |
| **Q3** | 0.98 | 0.70, 1.37 | 0.896 | 1.07 | 0.76, 1.52 | 0.693 | 1.01 | 0.72, 1.43 | 0.938 |
| **CHD** |  |  |  |  |  |  |  |  |  |
| **Q1** | Ref | Ref |  | Ref | Ref |  | Ref | Ref |  |
| **Q2** | 0.87 | 0.66, 1.15 | 0.335 | 0.88 | 0.66, 1.16 | 0.346 | 0.92 | 0.70, 1.22 | 0.574 |
| **Q3** | 0.89 | 0.68, 1.15 | 0.365 | 0.92 | 0.71, 1.20 | 0.549 | 0.99 | 0.75, 1.30 | 0.926 |
| **Angina** |  |  |  |  |  |  |  |  |  |
| **Q1** | Ref | Ref |  | Ref | Ref |  | Ref | Ref |  |
| **Q2** | 0.92 | 0.62, 1.36 | 0.681 | 0.96 | 0.64, 1.43 | 0.841 | 1.05 | 0.70, 1.58 | 0.817 |
| **Q3** | 1.02 | 0.70, 1.49 | 0.916 | 1.11 | 0.75, 1.64 | 0.599 | 1.21 | 0.80, 1.84 | 0.358 |
| **MI** |  |  |  |  |  |  |  |  |  |
| **Q1** | Ref | Ref |  | Ref | Ref |  | Ref | Ref |  |
| **Q2** | 0.97 | 0.72, 1.31 | 0.845 | 1.00 | 0.74, 1.36 | 0.992 | 1.04 | 0.74, 1.44 | 0.830 |
| **Q3** | 0.82 | 0.62, 1.08 | 0.157 | 0.87 | 0.65, 1.17 | 0.361 | 0.89 | 0.64, 1.23 | 0.479 |
| **Stroke** |  |  |  |  |  |  |  |  |  |
| **Q1** | Ref | Ref |  | Ref | Ref |  | Ref | Ref |  |
| **Q2** | 0.88 | 0.62, 1.23 | 0.443 | 0.93 | 0.67, 1.29 | 0.654 | 1.01 | 0.73, 1.42 | 0.931 |
| **Q3** | 0.76 | 0.53, 1.09 | 0.131 | 0.82 | 0.57, 1.17 | 0.269 | 0.90 | 0.62, 1.31 | 0.595 |

Supplemental Table 16

|  | **Model 1** | | | **Model 2** | | | **Model 3** | | |
| --- | --- | --- | --- | --- | --- | --- | --- | --- | --- |
|  | **HR1** | **95% CI1** | **p-value** | **HR1** | **95% CI1** | **p-value** | **HR1** | **95% CI1** | **p-value** |
|  | **DPA** |  |  |  |  |  |  |  |  |
| **ALL-cause** |  |  |  |  |  |  |  |  |  |
| **Q1** | Ref | Ref |  | Ref | Ref |  | Ref | Ref |  |
| **Q2** | 0.92 | 0.76, 1.12 | 0.400 | 0.96 | 0.79, 1.17 | 0.714 | 0.95 | 0.78, 1.16 | 0.600 |
| **Q3** | 0.73 | 0.61, 0.87 | <0.001** | 0.75 | 0.63, 0.90 | 0.002** | 0.76 | 0.65, 0.91 | 0.002** |
| **CVD** |  |  |  |  |  |  |  |  |  |
| **Q1** | Ref | Ref |  | Ref | Ref |  | Ref | Ref |  |
| **Q2** | 0.80 | 0.60, 1.07 | 0.134 | 0.82 | 0.61, 1.10 | 0.178 | 0.80 | 0.60, 1.08 | 0.149 |
| **Q3** | 0.68 | 0.51, 0.92 | 0.012* | 0.71 | 0.53, 0.94 | 0.019* | 0.70 | 0.52, 0.94 | 0.017* |
|  | **DHA** |  |  |  |  |  |  |  |  |
| **ALL-cause** |  |  |  |  |  |  |  |  |  |
| **Q1** | Ref | Ref |  | Ref | Ref |  | Ref | Ref |  |
| **Q2** | 1.07 | 0.91, 1.26 | 0.424 | 1.08 | 0.91, 1.28 | 0.398 | 1.10 | 0.92, 1.33 | 0.300 |
| **Q3** | 0.95 | 0.79, 1.15 | 0.604 | 0.94 | 0.77, 1.14 | 0.533 | 0.98 | 0.80, 1.20 | 0.823 |
| **CVD** |  |  |  |  |  |  |  |  |  |
| **Q1** | Ref | Ref |  | Ref | Ref |  | Ref | Ref |  |
| **Q2** | 0.99 | 0.77, 1.26 | 0.916 | 0.98 | 0.77, 1.27 | 0.905 | 1.00 | 0.74, 1.35 | 0.997 |
| **Q3** | 0.93 | 0.70, 1.24 | 0.639 | 0.93 | 0.69, 1.24 | 0.608 | 0.95 | 0.67, 1.34 | 0.768 |
|  | **EPA** |  |  |  |  |  |  |  |  |
| **ALL-cause** |  |  |  |  |  |  |  |  |  |
| **Q1** | Ref | Ref |  | Ref | Ref |  | Ref | Ref |  |
| **Q2** | 1.04 | 0.88, 1.24 | 0.630 | 1.07 | 0.90, 1.28 | 0.444 | 1.10 | 0.92, 1.32 | 0.283 |
| **Q3** | 0.85 | 0.72, 1.00 | 0.055 | 0.87 | 0.72, 1.04 | 0.118 | 0.89 | 0.75, 1.06 | 0.197 |
| **CVD** |  |  |  |  |  |  |  |  |  |
| **Q1** | Ref | Ref |  | Ref | Ref |  | Ref | Ref |  |
| **Q2** | 0.93 | 0.71, 1.22 | 0.619 | 0.94 | 0.71, 1.25 | 0.673 | 0.97 | 0.73, 1.29 | 0.837 |
| **Q3** | 0.85 | 0.66, 1.10 | 0.214 | 0.85 | 0.66, 1.11 | 0.245 | 0.87 | 0.66, 1.15 | 0.336 |

Supplemental Table 17

|  | **Model 1** | | | **Model 2** | | | **Model 3** | | |
| --- | --- | --- | --- | --- | --- | --- | --- | --- | --- |
|  | **OR^1^** | **95% CI^1^** | **p-value** | **OR^1^** | **95% CI^1^** | **p-value** | **OR^1^** | **95% CI^1^** | **p-value** |
| **CVD** |  |  |  |  |  |  |  |  |  |
| **Q1** | Ref | Ref |  | Ref | Ref |  | Ref | Ref |  |
| **Q2** | 0.83 | 0.69, 1.00 | 0.049* | 0.86 | 0.71, 1.03 | 0.100 | 0.76 | 0.62, 0.92 | 0.006** |
| **Q3** | 0.72 | 0.59, 0.88 | 0.002** | 0.75 | 0.61, 0.93 | 0.008** | 0.69 | 0.54, 0.87 | 0.002** |
| **CHF** |  |  |  |  |  |  |  |  |  |
| **Q1** | Ref | Ref |  | Ref | Ref |  | Ref | Ref |  |
| **Q2** | 0.86 | 0.66, 1.11 | 0.251 | 0.91 | 0.70, 1.19 | 0.480 | 0.78 | 0.58, 1.05 | 0.103 |
| **Q3** | 0.88 | 0.65, 1.18 | 0.378 | 0.94 | 0.69, 1.29 | 0.698 | 0.83 | 0.59, 1.18 | 0.303 |
| **CHD** |  |  |  |  |  |  |  |  |  |
| **Q1** | Ref | Ref |  | Ref | Ref |  | Ref | Ref |  |
| **Q2** | 0.85 | 0.67, 1.07 | 0.166 | 0.86 | 0.69, 1.08 | 0.192 | 0.80 | 0.64, 1.00 | 0.050 |
| **Q3** | 0.78 | 0.61, 0.99 | 0.044* | 0.80 | 0.63, 1.03 | 0.085 | 0.80 | 0.60, 1.06 | 0.122 |
| **Angina** |  |  |  |  |  |  |  |  |  |
| **Q1** | Ref | Ref |  | Ref | Ref |  | Ref | Ref |  |
| **Q2** | 0.74 | 0.53, 1.01 | 0.061 | 0.75 | 0.54, 1.03 | 0.078 | 0.67 | 0.48, 0.94 | 0.019* |
| **Q3** | 0.63 | 0.45, 0.88 | 0.007** | 0.65 | 0.46, 0.91 | 0.014* | 0.55 | 0.39, 0.78 | <0.001** |
| **MI** |  |  |  |  |  |  |  |  |  |
| **Q1** | Ref | Ref |  | Ref | Ref |  | Ref | Ref |  |
| **Q2** | 0.90 | 0.70, 1.15 | 0.378 | 0.94 | 0.73, 1.20 | 0.592 | 0.86 | 0.66, 1.11 | 0.230 |
| **Q3** | 0.81 | 0.62, 1.04 | 0.096 | 0.85 | 0.65, 1.12 | 0.253 | 0.79 | 0.60, 1.04 | 0.091 |
| **Stroke** |  |  |  |  |  |  |  |  |  |
| **Q1** | Ref | Ref |  | Ref | Ref |  | Ref | Ref |  |
| **Q2** | 0.80 | 0.58, 1.10 | 0.160 | 0.83 | 0.60, 1.15 | 0.260 | 0.79 | 0.56, 1.11 | 0.174 |
| **Q3** | 0.63 | 0.45, 0.87 | 0.006** | 0.66 | 0.48, 0.92 | 0.015* | 0.61 | 0.41, 0.91 | 0.016* |

Supplemental Table 18

|  | **Model 1** | | | **Model 2** | | | **Model 3** | | |
| --- | --- | --- | --- | --- | --- | --- | --- | --- | --- |
|  | **OR^1^** | **95% CI^1^** | **p-value** | **OR^1^** | **95% CI^1^** | **p-value** | **OR^1^** | **95% CI^1^** | **p-value** |
| **CVD** |  |  |  |  |  |  |  |  |  |
| **Q1** | Ref | Ref |  | Ref | Ref |  | Ref | Ref |  |
| **Q2** | 0.92 | 0.77, 1.11 | 0.405 | 0.94 | 0.77, 1.14 | 0.500 | 0.93 | 0.74, 1.18 | 0.554 |
| **Q3** | 0.92 | 0.77, 1.09 | 0.312 | 0.96 | 0.80, 1.15 | 0.660 | 0.96 | 0.76, 1.22 | 0.760 |
| **CHF** |  |  |  |  |  |  |  |  |  |
| **Q1** | Ref | Ref |  | Ref | Ref |  | Ref | Ref |  |
| **Q2** | 1.03 | 0.78, 1.36 | 0.819 | 1.05 | 0.80, 1.39 | 0.699 | 1.05 | 0.77, 1.43 | 0.744 |
| **Q3** | 0.99 | 0.74, 1.33 | 0.965 | 1.05 | 0.77, 1.44 | 0.745 | 0.99 | 0.68, 1.45 | 0.956 |
| **CHD** |  |  |  |  |  |  |  |  |  |
| **Q1** | Ref | Ref |  | Ref | Ref |  | Ref | Ref |  |
| **Q2** | 0.86 | 0.68, 1.09 | 0.201 | 0.85 | 0.67, 1.08 | 0.183 | 0.94 | 0.72, 1.23 | 0.655 |
| **Q3** | 0.95 | 0.77, 1.18 | 0.645 | 0.97 | 0.78, 1.21 | 0.791 | 1.11 | 0.85, 1.44 | 0.436 |
| **Angina** |  |  |  |  |  |  |  |  |  |
| **Q1** | Ref | Ref |  | Ref | Ref |  | Ref | Ref |  |
| **Q2** | 0.84 | 0.62, 1.15 | 0.277 | 0.85 | 0.62, 1.17 | 0.328 | 0.92 | 0.65, 1.31 | 0.659 |
| **Q3** | 1.00 | 0.72, 1.39 | 0.997 | 1.05 | 0.74, 1.48 | 0.787 | 1.06 | 0.73, 1.53 | 0.763 |
| **MI** |  |  |  |  |  |  |  |  |  |
| **Q1** | Ref | Ref |  | Ref | Ref |  | Ref | Ref |  |
| **Q2** | 1.02 | 0.84, 1.24 | 0.866 | 1.03 | 0.84, 1.26 | 0.782 | 1.02 | 0.79, 1.32 | 0.891 |
| **Q3** | 1.02 | 0.82, 1.27 | 0.833 | 1.07 | 0.84, 1.36 | 0.568 | 1.02 | 0.77, 1.34 | 0.909 |
| **Stroke** |  |  |  |  |  |  |  |  |  |
| **Q1** | Ref | Ref |  | Ref | Ref |  | Ref | Ref |  |
| **Q2** | 0.85 | 0.63, 1.16 | 0.302 | 0.86 | 0.63, 1.18 | 0.362 | 0.76 | 0.52, 1.13 | 0.180 |
| **Q3** | 0.77 | 0.56, 1.06 | 0.108 | 0.82 | 0.59, 1.13 | 0.217 | 0.75 | 0.47, 1.20 | 0.230 |

Supplemental Table 19

|  | **Model 1** | | | **Model 2** | | | **Model 3** | | |
| --- | --- | --- | --- | --- | --- | --- | --- | --- | --- |
|  | **OR^1^** | **95% CI^1^** | **p-value** | **OR^1^** | **95% CI^1^** | **p-value** | **OR^1^** | **95% CI^1^** | **p-value** |
| **CVD** |  |  |  |  |  |  |  |  |  |
| **Q1** | Ref | Ref |  | Ref | Ref |  | Ref | Ref |  |
| **Q2** | 0.87 | 0.72, 1.05 | 0.150 | 0.92 | 0.76, 1.11 | 0.369 | 0.89 | 0.73, 1.09 | 0.277 |
| **Q3** | 0.72 | 0.59, 0.88 | 0.002** | 0.84 | 0.68, 1.04 | 0.102 | 0.81 | 0.65, 1.02 | 0.079 |
| **CHF** |  |  |  |  |  |  |  |  |  |
| **Q1** | Ref | Ref |  | Ref | Ref |  | Ref | Ref |  |
| **Q2** | 0.66 | 0.48, 0.90 | 0.010* | 0.69 | 0.49, 0.97 | 0.031* | 0.65 | 0.46, 0.91 | 0.014* |
| **Q3** | 0.53 | 0.36, 0.78 | 0.001** | 0.63 | 0.42, 0.94 | 0.025* | 0.60 | 0.40, 0.89 | 0.012* |
| **CHD** |  |  |  |  |  |  |  |  |  |
| **Q1** | Ref | Ref |  | Ref | Ref |  | Ref | Ref |  |
| **Q2** | 0.86 | 0.62, 1.18 | 0.337 | 0.92 | 0.66, 1.28 | 0.622 | 0.93 | 0.66, 1.31 | 0.674 |
| **Q3** | 0.71 | 0.52, 0.96 | 0.025* | 0.84 | 0.62, 1.14 | 0.264 | 0.85 | 0.62, 1.16 | 0.302 |
| **Angina** |  |  |  |  |  |  |  |  |  |
| **Q1** | Ref | Ref |  | Ref | Ref |  | Ref | Ref |  |
| **Q2** | 0.67 | 0.49, 0.93 | 0.017* | 0.71 | 0.51, 0.99 | 0.041* | 0.74 | 0.52, 1.05 | 0.090 |
| **Q3** | 0.69 | 0.51, 0.95 | 0.022* | 0.82 | 0.59, 1.12 | 0.205 | 0.87 | 0.62, 1.23 | 0.424 |
| **MI** |  |  |  |  |  |  |  |  |  |
| **Q1** | Ref | Ref |  | Ref | Ref |  | Ref | Ref |  |
| **Q2** | 0.73 | 0.54, 0.98 | 0.037* | 0.76 | 0.56, 1.03 | 0.076 | 0.82 | 0.60, 1.12 | 0.211 |
| **Q3** | 0.86 | 0.63, 1.19 | 0.369 | 1.02 | 0.74, 1.41 | 0.916 | 1.13 | 0.81, 1.57 | 0.483 |
| **Stroke** |  |  |  |  |  |  |  |  |  |
| **Q1** | Ref | Ref |  | Ref | Ref |  | Ref | Ref |  |
| **Q2** | 0.97 | 0.75, 1.25 | 0.812 | 1.02 | 0.79, 1.31 | 0.890 | 0.97 | 0.74, 1.26 | 0.812 |
| **Q3** | 0.70 | 0.54, 0.92 | 0.011* | 0.81 | 0.61, 1.07 | 0.129 | 0.75 | 0.54, 1.04 | 0.085 |

Supplemental Table 20

|  | **Model 1** | | | **Model 2** | | | **Model 3** | | |
| --- | --- | --- | --- | --- | --- | --- | --- | --- | --- |
|  | **HR1** | **95% CI1** | **p-value** | **HR1** | **95% CI1** | **p-value** | **HR1** | **95% CI1** | **p-value** |
|  | **DPA** |  |  |  |  |  |  |  |  |
| **ALL-cause** |  |  |  |  |  |  |  |  |  |
| **Q1** | Ref | Ref |  | Ref | Ref |  | Ref | Ref |  |
| **Q2** | 0.80 | 0.70, 0.93 | 0.002** | 0.85 | 0.74, 0.98 | 0.029* | 0.85 | 0.75, 0.98 | 0.021* |
| **Q3** | 0.70 | 0.61, 0.80 | <0.001** | 0.74 | 0.64, 0.86 | <0.001** | 0.77 | 0.66, 0.91 | 0.002** |
| **CVD** |  |  |  |  |  |  |  |  |  |
| **Q1** | Ref | Ref |  | Ref | Ref |  | Ref | Ref |  |
| **Q2** | 0.77 | 0.59, 1.01 | 0.062 | 0.83 | 0.63, 1.08 | 0.168 | 0.78 | 0.61, 1.01 | 0.060 |
| **Q3** | 0.62 | 0.49, 0.80 | <0.001** | 0.67 | 0.52, 0.87 | 0.002** | 0.71 | 0.55, 0.92 | 0.011* |
|  | **DHA** |  |  |  |  |  |  |  |  |
| **ALL-cause** |  |  |  |  |  |  |  |  |  |
| **Q1** | Ref | Ref |  | Ref | Ref |  | Ref | Ref |  |
| **Q2** | 1.12 | 0.99, 1.27 | 0.068 | 1.16 | 1.02, 1.31 | 0.019* | 1.09 | 0.95, 1.25 | 0.226 |
| **Q3** | 0.89 | 0.77, 1.03 | 0.116 | 0.94 | 0.80, 1.10 | 0.433 | 0.94 | 0.79, 1.14 | 0.544 |
| **CVD** |  |  |  |  |  |  |  |  |  |
| **Q1** | Ref | Ref |  | Ref | Ref |  | Ref | Ref |  |
| **Q2** | 1.11 | 0.92, 1.33 | 0.275 | 1.15 | 0.95, 1.39 | 0.158 | 1.07 | 0.86, 1.33 | 0.546 |
| **Q3** | 0.80 | 0.67, 0.96 | 0.018* | 0.84 | 0.68, 1.03 | 0.094 | 0.88 | 0.67, 1.15 | 0.355 |
|  | **EPA** |  |  |  |  |  |  |  |  |
| **ALL-cause** |  |  |  |  |  |  |  |  |  |
| **Q1** | Ref | Ref |  | Ref | Ref |  | Ref | Ref |  |
| **Q2** | 0.85 | 0.74, 0.96 | 0.011* | 0.88 | 0.77, 1.00 | 0.042* | 0.86 | 0.75, 0.99 | 0.030* |
| **Q3** | 0.78 | 0.67, 0.91 | 0.001** | 0.84 | 0.71, 0.99 | 0.035* | 0.87 | 0.74, 1.03 | 0.106 |
| **CVD** |  |  |  |  |  |  |  |  |  |
| **Q1** | Ref | Ref |  | Ref | Ref |  | Ref | Ref |  |
| **Q2** | 0.83 | 0.65, 1.07 | 0.155 | 0.86 | 0.67, 1.12 | 0.260 | 0.82 | 0.64, 1.05 | 0.121 |
| **Q3** | 0.74 | 0.59, 0.93 | 0.010* | 0.80 | 0.63, 1.01 | 0.056 | 0.82 | 0.64, 1.05 | 0.117 |

Supplemental Table 21

|  | **Model 1** | | | **Model 2** | | | **Model 3** | | |
| --- | --- | --- | --- | --- | --- | --- | --- | --- | --- |
|  | **OR^1^** | **95% CI^1^** | **p-value** | **OR^1^** | **95% CI^1^** | **p-value** | **OR^1^** | **95% CI^1^** | **p-value** |
| **CVD** |  |  |  |  |  |  |  |  |  |
| **Q1** | Ref | Ref |  | Ref | Ref |  | Ref | Ref |  |
| **Q2** | 0.82 | 0.67, 1.00 | 0.047* | 0.87 | 0.71, 1.07 | 0.177 | 0.84 | 0.67, 1.04 | 0.104 |
| **Q3** | 0.85 | 0.70, 1.04 | 0.110 | 0.96 | 0.79, 1.17 | 0.704 | 0.92 | 0.73, 1.17 | 0.513 |
| **CHF** |  |  |  |  |  |  |  |  |  |
| **Q1** | Ref | Ref |  | Ref | Ref |  | Ref | Ref |  |
| **Q2** | 0.8 | 0.58, 1.09 | 0.156 | 0.82 | 0.59, 1.15 | 0.252 | 0.78 | 0.56, 1.08 | 0.127 |
| **Q3** | 0.83 | 0.59, 1.17 | 0.275 | 0.95 | 0.66, 1.36 | 0.760 | 0.88 | 0.60, 1.29 | 0.506 |
| **CHD** |  |  |  |  |  |  |  |  |  |
| **Q1** | Ref | Ref |  | Ref | Ref |  | Ref | Ref |  |
| **Q2** | 0.94 | 0.67, 1.31 | 0.699 | 1.03 | 0.73, 1.46 | 0.847 | 1.03 | 0.72, 1.46 | 0.871 |
| **Q3** | 0.82 | 0.60, 1.13 | 0.224 | 0.97 | 0.70, 1.33 | 0.829 | 0.96 | 0.69, 1.33 | 0.795 |
| **Angina** |  |  |  |  |  |  |  |  |  |
| **Q1** | Ref | Ref |  | Ref | Ref |  | Ref | Ref |  |
| **Q2** | 0.71 | 0.50, 1.02 | 0.062 | 0.76 | 0.53, 1.11 | 0.152 | 0.76 | 0.52, 1.11 | 0.151 |
| **Q3** | 0.68 | 0.52, 0.89 | 0.006** | 0.77 | 0.58, 1.02 | 0.067 | 0.81 | 0.58, 1.14 | 0.230 |
| **MI** |  |  |  |  |  |  |  |  |  |
| **Q1** | Ref | Ref |  | Ref | Ref |  | Ref | Ref |  |
| **Q2** | 0.92 | 0.67, 1.24 | 0.571 | 0.99 | 0.71, 1.38 | 0.960 | 1.02 | 0.72, 1.45 | 0.889 |
| **Q3** | 1.03 | 0.76, 1.41 | 0.837 | 1.18 | 0.87, 1.60 | 0.282 | 1.35 | 0.98, 1.85 | 0.062 |
| **Stroke** |  |  |  |  |  |  |  |  |  |
| **Q1** | Ref | Ref |  | Ref | Ref |  | Ref | Ref |  |
| **Q2** | 0.76 | 0.57, 1.02 | 0.069 | 0.80 | 0.60, 1.08 | 0.141 | 0.77 | 0.56, 1.05 | 0.097 |
| **Q3** | 0.87 | 0.63, 1.20 | 0.386 | 0.97 | 0.70, 1.33 | 0.844 | 0.89 | 0.60, 1.32 | 0.560 |

Supplemental Table 22

|  | **Model 1** | | | **Model 2** | | | **Model 3** | | |
| --- | --- | --- | --- | --- | --- | --- | --- | --- | --- |
|  | **OR^1^** | **95% CI^1^** | **p-value** | **OR^1^** | **95% CI^1^** | **p-value** | **OR^1^** | **95% CI^1^** | **p-value** |
| **CVD** |  |  |  |  |  |  |  |  |  |
| **Q1** | Ref | Ref |  | Ref | Ref |  | Ref | Ref |  |
| **Q2** | 0.90 | 0.74, 1.09 | 0.279 | 0.94 | 0.76, 1.16 | 0.542 | 0.91 | 0.72, 1.15 | 0.422 |
| **Q3** | 0.78 | 0.64, 0.95 | 0.015 | 0.89 | 0.73, 1.08 | 0.232 | 0.83 | 0.65, 1.06 | 0.137 |
| **CHF** |  |  |  |  |  |  |  |  |  |
| **Q1** | Ref | Ref |  | Ref | Ref |  | Ref | Ref |  |
| **Q2** | 0.93 | 0.65, 1.32 | 0.665 | 0.96 | 0.68, 1.37 | 0.835 | 0.9 | 0.62, 1.29 | 0.561 |
| **Q3** | 0.75 | 0.53, 1.05 | 0.093 | 0.87 | 0.61, 1.25 | 0.453 | 0.77 | 0.54, 1.10 | 0.144 |
| **CHD** |  |  |  |  |  |  |  |  |  |
| **Q1** | Ref | Ref |  | Ref | Ref |  | Ref | Ref |  |
| **Q2** | 0.95 | 0.69, 1.32 | 0.773 | 1.00 | 0.72, 1.38 | 0.976 | 1.08 | 0.74, 1.57 | 0.682 |
| **Q3** | 0.65 | 0.47, 0.90 | 0.010* | 0.74 | 0.53, 1.03 | 0.074 | 0.79 | 0.56, 1.13 | 0.192 |
| **Angina** |  |  |  |  |  |  |  |  |  |
| **Q1** | Ref | Ref |  | Ref | Ref |  | Ref | Ref |  |
| **Q2** | 1.03 | 0.74, 1.45 | 0.851 | 1.08 | 0.77, 1.51 | 0.652 | 1.21 | 0.86, 1.70 | 0.274 |
| **Q3** | 0.76 | 0.57, 1.03 | 0.073 | 0.87 | 0.64, 1.17 | 0.354 | 1.00 | 0.69, 1.46 | 0.980 |
| **MI** |  |  |  |  |  |  |  |  |  |
| **Q1** | Ref | Ref |  | Ref | Ref |  | Ref | Ref |  |
| **Q2** | 0.83 | 0.63, 1.09 | 0.176 | 0.86 | 0.66, 1.13 | 0.281 | 1.00 | 0.74, 1.35 | 0.998 |
| **Q3** | 0.82 | 0.62, 1.09 | 0.174 | 0.93 | 0.70, 1.23 | 0.598 | 1.14 | 0.86, 1.51 | 0.360 |
| **Stroke** |  |  |  |  |  |  |  |  |  |
| **Q1** | Ref | Ref |  | Ref | Ref |  | Ref | Ref |  |
| **Q2** | 0.91 | 0.67, 1.23 | 0.528 | 0.95 | 0.69, 1.31 | 0.745 | 0.84 | 0.56, 1.26 | 0.386 |
| **Q3** | 0.76 | 0.58, 1.00 | 0.051 | 0.86 | 0.65, 1.13 | 0.277 | 0.70 | 0.47, 1.05 | 0.084 |

Supplemental Table 23

|  | **Model 1** | | | **Model 2** | | | **Model 3** | | |
| --- | --- | --- | --- | --- | --- | --- | --- | --- | --- |
|  | **OR^1^** | **95% CI^1^** | **p-value** | **OR^1^** | **95% CI^1^** | **p-value** | **OR^1^** | **95% CI^1^** | **p-value** |
| **CVD** |  |  |  |  |  |  |  |  |  |
| **Q1** | Ref | Ref |  | Ref | Ref |  | Ref | Ref |  |
| **Q2** | 0.93 | 0.78, 1.12 | 0.440 | 0.98 | 0.81, 1.17 | 0.786 | 0.92 | 0.75, 1.13 | 0.411 |
| **Q3** | 0.86 | 0.72, 1.03 | 0.094 | 0.95 | 0.78, 1.16 | 0.599 | 0.93 | 0.74, 1.17 | 0.552 |
| **CHF** |  |  |  |  |  |  |  |  |  |
| **Q1** | Ref | Ref |  | Ref | Ref |  | Ref | Ref |  |
| **Q2** | 1.12 | 0.88, 1.41 | 0.345 | 1.20 | 0.95, 1.51 | 0.128 | 1.11 | 0.87, 1.41 | 0.411 |
| **Q3** | 0.90 | 0.68, 1.21 | 0.492 | 1.03 | 0.76, 1.41 | 0.838 | 0.97 | 0.71, 1.32 | 0.842 |
| **CHD** |  |  |  |  |  |  |  |  |  |
| **Q1** | Ref | Ref |  | Ref | Ref |  | Ref | Ref |  |
| **Q2** | 0.85 | 0.68, 1.06 | 0.153 | 0.87 | 0.70, 1.10 | 0.241 | 0.89 | 0.70, 1.12 | 0.311 |
| **Q3** | 1.00 | 0.82, 1.22 | 0.978 | 1.07 | 0.86, 1.32 | 0.536 | 1.13 | 0.89, 1.44 | 0.304 |
| **Angina** |  |  |  |  |  |  |  |  |  |
| **Q1** | Ref | Ref |  | Ref | Ref |  | Ref | Ref |  |
| **Q2** | 0.94 | 0.70, 1.27 | 0.682 | 0.98 | 0.72, 1.35 | 0.917 | 0.93 | 0.67, 1.30 | 0.684 |
| **Q3** | 0.91 | 0.68, 1.22 | 0.520 | 1.01 | 0.74, 1.38 | 0.949 | 0.95 | 0.66, 1.36 | 0.773 |
| **MI** |  |  |  |  |  |  |  |  |  |
| **Q1** | Ref | Ref |  | Ref | Ref |  | Ref | Ref |  |
| **Q2** | 0.97 | 0.78, 1.21 | 0.801 | 1.03 | 0.82, 1.28 | 0.816 | 1.00 | 0.79, 1.27 | 0.993 |
| **Q3** | 0.86 | 0.68, 1.09 | 0.206 | 0.96 | 0.75, 1.24 | 0.753 | 0.93 | 0.71, 1.22 | 0.606 |
| **Stroke** |  |  |  |  |  |  |  |  |  |
| **Q1** | Ref | Ref |  | Ref | Ref |  | Ref | Ref |  |
| **Q2** | 0.70 | 0.52, 0.95 | 0.023* | 0.73 | 0.53, 0.99 | 0.041* | 0.67 | 0.46, 1.00 | 0.049* |
| **Q3** | 0.65 | 0.47, 0.89 | 0.009** | 0.72 | 0.52, 1.00 | 0.048* | 0.70 | 0.46, 1.05 | 0.083 |

Supplemental Table 24

|  | **Model 1** | | | **Model 2** | | | **Model 3** | | |
| --- | --- | --- | --- | --- | --- | --- | --- | --- | --- |
|  | **HR1** | **95% CI1** | **p-value** | **HR1** | **95% CI1** | **p-value** | **HR1** | **95% CI1** | **p-value** |
|  | **DPA** |  |  |  |  |  |  |  |  |
| **ALL-cause** |  |  |  |  |  |  |  |  |  |
| **Q1** | Ref | Ref |  | Ref | Ref |  | Ref | Ref |  |
| **Q2** | 0.81 | 0.70, 0.95 | 0.009** | 0.87 | 0.75, 1.01 | 0.065 | 0.85 | 0.72, 0.99 | 0.040* |
| **Q3** | 0.79 | 0.65, 0.95 | 0.011* | 0.89 | 0.74, 1.07 | 0.217 | 0.87 | 0.72, 1.06 | 0.175 |
| **CVD** |  |  |  |  |  |  |  |  |  |
| **Q1** | Ref | Ref |  | Ref | Ref |  | Ref | Ref |  |
| **Q2** | 0.70 | 0.53, 0.93 | 0.012* | 0.75 | 0.58, 0.98 | 0.033* | 0.73 | 0.56, 0.97 | 0.027* |
| **Q3** | 0.76 | 0.56, 1.03 | 0.074 | 0.89 | 0.66, 1.20 | 0.457 | 0.86 | 0.62, 1.20 | 0.366 |
|  | **DHA** |  |  |  |  |  |  |  |  |
| **ALL-cause** |  |  |  |  |  |  |  |  |  |
| **Q1** | Ref | Ref |  | Ref | Ref |  | Ref | Ref |  |
| **Q2** | 0.80 | 0.69, 0.93 | 0.003** | 0.87 | 0.75, 1.00 | 0.049* | 0.81 | 0.69, 0.96 | 0.017* |
| **Q3** | 0.81 | 0.70, 0.93 | 0.002** | 0.94 | 0.81, 1.08 | 0.376 | 0.87 | 0.73, 1.05 | 0.141 |
| **CVD** |  |  |  |  |  |  |  |  |  |
| **Q1** | Ref | Ref |  | Ref | Ref |  | Ref | Ref |  |
| **Q2** | 0.82 | 0.66, 1.03 | 0.083 | 0.90 | 0.74, 1.10 | 0.314 | 0.78 | 0.60, 1.02 | 0.066 |
| **Q3** | 0.88 | 0.68, 1.14 | 0.335 | 1.08 | 0.83, 1.39 | 0.576 | 0.91 | 0.67, 1.25 | 0.562 |
|  | **EPA** |  |  |  |  |  |  |  |  |
| **ALL-cause** |  |  |  |  |  |  |  |  |  |
| **Q1** | Ref | Ref |  | Ref | Ref |  | Ref | Ref |  |
| **Q2** | 0.88 | 0.76, 1.01 | 0.060 | 0.95 | 0.83, 1.09 | 0.469 | 0.93 | 0.81, 1.08 | 0.333 |
| **Q3** | 0.78 | 0.68, 0.91 | 0.001** | 0.93 | 0.78, 1.10 | 0.405 | 0.91 | 0.76, 1.08 | 0.286 |
| **CVD** |  |  |  |  |  |  |  |  |  |
| **Q1** | Ref | Ref |  | Ref | Ref |  | Ref | Ref |  |
| **Q2** | 0.87 | 0.66, 1.14 | 0.314 | 0.98 | 0.76, 1.26 | 0.855 | 0.93 | 0.72, 1.19 | 0.552 |
| **Q3** | 0.73 | 0.58, 0.93 | 0.009** | 0.92 | 0.71, 1.19 | 0.512 | 0.87 | 0.67, 1.12 | 0.284 |

Supplemental Table 1

^1^OR: odds ratio, CI: confidence interval; CHF: congestive heart failure; CHD: coronary heart disease; MI: myocardial infarction; **P*<0.05; ***P*<0.01; Multivariate adjusted Logistic regression models of DPA intake with cardiovascular disease in overweight or obese people: Model 1: Age and race; Model 2: Model 1+Education, marital status, ratio of family income to poverty, BMI, physical activity, smoking status and alcohol consumption；Model 3： Model 2+hypertension, diabetes, cancer, osteoarthritis, total energy intake, total fat intake, and total cholesterol intake.

Supplemental Table 2

^1^OR: odds ratio, CI: confidence interval; CHF: congestive heart failure; CHD: coronary heart disease; MI: myocardial infarction; Multivariate adjusted Logistic regression models of DHA intake with cardiovascular disease in overweight or obese people: Model 1: Age and race; Model 2: Model 1+Education, marital status, ratio of family income to poverty, BMI, physical activity, smoking status and alcohol consumption；Model 3： Model 2+hypertension, diabetes, cancer, osteoarthritis, total energy intake, total fat intake, and total cholesterol intake.

Supplemental Table 3

^1^OR: odds ratio, CI: confidence interval; CHF: congestive heart failure; CHD: coronary heart disease; MI: myocardial infarction; **P*<0.05; ***P*<0.01; Multivariate adjusted Logistic regression models of EPA intake with cardiovascular disease in overweight or obese people: Model 1: Age and race; Model 2: Model 1+Education, marital status, ratio of family income to poverty, BMI, physical activity, smoking status and alcohol consumption；Model 3： Model 2+hypertension, diabetes, cancer, osteoarthritis, total energy intake, total fat intake, and total cholesterol intake.

Supplemental Table 4

^1^HR: hazard ratio, CI: confidence interval; CVD: CVD mortality; ALL-cause: All-cause mortality; **P*<0.05; ***P*<0.01; Multivariate adjusted Cox proportional regression models of marine polyunsaturated fatty acids intake with cardiovascular disease in overweight or obese people: Model 1: Age and race; Model 2：Model 1+education, marital status, ratio of family income to poverty, BMI, physical activity, smoking status and alcohol consumption；Model 3：Model 2+hypertension, diabetes, cancer, osteoarthritis, total energy intake, total fat intake, and total cholesterol intake.

Supplemental Table 5

^1^OR: odds ratio, CI: confidence interval; CHF: congestive heart failure; CHD: coronary heart disease; MI: myocardial infarction; **P*<0.05; ***P*<0.01; Multivariate adjusted Logistic regression models of DPA intake with cardiovascular disease in a population followed for more than two years: Model 1: Age and race; Model 2: Model 1+Education, marital status, ratio of family income to poverty, BMI, physical activity, smoking status and alcohol consumption；Model 3： Model 2+hypertension, diabetes, cancer, osteoarthritis, total energy intake, total fat intake, and total cholesterol intake.

Supplemental Table 6

^1^OR: odds ratio, CI: confidence interval; CHF: congestive heart failure; CHD: coronary heart disease; MI: myocardial infarction; **P*<0.05; Multivariate adjusted Logistic regression models of DHA intake with cardiovascular disease in a population followed for more than two years: Model 1: Age and race; Model 2: Model 1+Education, marital status, ratio of family income to poverty, BMI, physical activity, smoking status and alcohol consumption；Model 3： Model 2+hypertension, diabetes, cancer, osteoarthritis, total energy intake, total fat intake, and total cholesterol intake.

Supplemental Table 7

^1^OR: odds ratio, CI: confidence interval; CHF: congestive heart failure; CHD: coronary heart disease; MI: myocardial infarction; **P*<0.05; ***P*<0.01; Multivariate adjusted Logistic regression models of EPA intake with cardiovascular disease in a population followed for more than two years: Model 1: Age and race; Model 2: Model 1+Education, marital status, ratio of family income to poverty, BMI, physical activity, smoking status and alcohol consumption；Model 3： Model 2+hypertension, diabetes, cancer, osteoarthritis, total energy intake, total fat intake, and total cholesterol intake.

Supplemental Table 8

^1^HR: hazard ratio, CI: confidence interval; CVD: CVD mortality; ALL-cause: All-cause mortality; **P*<0.05; ***P*<0.01; Multivariate adjusted Cox proportional regression models of marine polyunsaturated fatty acids intake with cardiovascular disease in a population followed for more than two years: Model 1: Age and race; Model 2：Model 1+education, marital status, ratio of family income to poverty, BMI, physical activity, smoking status and alcohol consumption；Model 3：Model 2+hypertension, diabetes, cancer, osteoarthritis, total energy intake, total fat intake, and total cholesterol intake.

Supplemental Table 9

^1^OR: odds ratio, CI: confidence interval; CHF: congestive heart failure; CHD: coronary heart disease; MI: myocardial infarction; **P*<0.05; ***P*<0.01; Multivariate adjusted Logistic regression models of DPA intake with cardiovascular disease in a population without extreme dietary fat intake: Model 1: Age and race; Model 2: Model 1+Education, marital status, ratio of family income to poverty, BMI, physical activity, smoking status and alcohol consumption；Model 3： Model 2+hypertension, diabetes, cancer, osteoarthritis, total energy intake, total fat intake, and total cholesterol intake.

Supplemental Table 10

^1^OR: odds ratio, CI: confidence interval; CHF: congestive heart failure; CHD: coronary heart disease; MI: myocardial infarction; **P*<0.05; Multivariate adjusted Logistic regression models of DHA intake with cardiovascular disease in a population without extreme dietary fat intake: Model 1: Age and race; Model 2: Model 1+Education, marital status, ratio of family income to poverty, BMI, physical activity, smoking status and alcohol consumption；Model 3： Model 2+hypertension, diabetes, cancer, osteoarthritis, total energy intake, total fat intake, and total cholesterol intake.

Supplemental Table 11

^1^OR: odds ratio, CI: confidence interval; CHF: congestive heart failure; CHD: coronary heart disease; MI: myocardial infarction; **P*<0.05; ***P*<0.01; Multivariate adjusted Logistic regression models of EPA intake with cardiovascular disease in a population without extreme dietary fat intake: Model 1: Age and race; Model 2: Model 1+Education, marital status, ratio of family income to poverty, BMI, physical activity, smoking status and alcohol consumption；Model 3： Model 2+hypertension, diabetes, cancer, osteoarthritis, total energy intake, total fat intake, and total cholesterol intake.

Supplemental Table 12

^1^HR: hazard ratio, CI: confidence interval; CVD: CVD mortality; ALL-cause: All-cause mortality; **P*<0.05; ***P*<0.01; Multivariate adjusted Cox proportional regression models of marine polyunsaturated fatty acids intake with cardiovascular disease in a population without extreme dietary fat intake: Model 1: Age and race; Model 2：Model 1+education, marital status, ratio of family income to poverty, BMI, physical activity, smoking status and alcohol consumption；Model 3：Model 2+hypertension, diabetes, cancer, osteoarthritis, total energy intake, total fat intake, and total cholesterol intake.

Supplemental Table 13

^1^OR: odds ratio, CI: confidence interval; CHF: congestive heart failure; CHD: coronary heart disease; MI: myocardial infarction; **P*<0.05; ***P*<0.01; Multivariate adjusted Logistic regression models of DPA intake with cardiovascular disease in an elderly population: Model 1: Race; Model 2: Model 1+Education, Marital status, Ratio of family income to poverty, BMI, Physical activity, Smoking status and Alcohol consumption； Model 3： Model 2+Hypertension, diabetes, cancer, osteoarthritis, total energy intake, total fat intake, and total cholesterol intake.

Supplemental Table 14

^1^OR: odds ratio, CI: confidence interval; CHF: congestive heart failure; CHD: coronary heart disease; MI: myocardial infarction; Multivariate adjusted Logistic regression models of DHA intake with cardiovascular disease in an elderly population: Model 1: Race; Model 2: Model 1+Education, Marital status, Ratio of family income to poverty, BMI, Physical activity, Smoking status and Alcohol consumption； Model 3： Model 2+Hypertension, diabetes, cancer, osteoarthritis, total energy intake, total fat intake, and total cholesterol intake.

Supplemental Table 15

^1^OR: odds ratio, CI: confidence interval; CHF: congestive heart failure; CHD: coronary heart disease; MI: myocardial infarction; Multivariate adjusted Logistic regression models of EPA intake with cardiovascular disease in an elderly population: Model 1: Race; Model 2: Model 1+Education, Marital status, Ratio of family income to poverty, BMI, Physical activity, Smoking status and Alcohol consumption； Model 3： Model 2+Hypertension, diabetes, cancer, osteoarthritis, total energy intake, total fat intake, and total cholesterol intake.

Supplemental Table 16

^1^HR: hazard ratio, CI: confidence interval; CVD: CVD mortality; ALL-cause: All-cause mortality; **P*<0.05; ***P*<0.01; Multivariate adjusted Cox proportional regression models of marine polyunsaturated fatty acids intake with cardiovascular disease in an elderly population: Model 1: Race; Model 2：Model 1+education, marital status, ratio of family income to poverty, BMI, physical activity, smoking status and alcohol consumption；Model 3：Model 2+hypertension, diabetes, cancer, osteoarthritis, total energy intake, total fat intake, and total cholesterol intake.

Supplemental Table 17

^1^OR: odds ratio, CI: confidence interval; CHF: congestive heart failure; CHD: coronary heart disease; MI: myocardial infarction; **P*<0.05; ***P*<0.01; Multivariate adjusted Logistic regression models of DPA intake with cardiovascular disease in women with dyslipidemia: Model 1: Age and race; Model 2: Model 1+Education, Marital status, Ratio of family income to poverty, BMI, Physical activity, Smoking status and Alcohol consumption； Model 3： Model 2+Hypertension, diabetes, cancer, osteoarthritis, total energy intake, total fat intake, and total cholesterol intake.

Supplemental Table 18

^1^OR: odds ratio, CI: confidence interval; CHF: congestive heart failure; CHD: coronary heart disease; MI: myocardial infarction; Multivariate adjusted Logistic regression models of DHA intake with cardiovascular disease in women with dyslipidemia: Model 1: Age and race; Model 2: Model 1+Education, Marital status, Ratio of family income to poverty, BMI, Physical activity, Smoking status and Alcohol consumption； Model 3： Model 2+Hypertension, diabetes, cancer, osteoarthritis, total energy intake, total fat intake, and total cholesterol intake.

Supplemental Table 19

^1^OR: odds ratio, CI: confidence interval; CHF: congestive heart failure; CHD: coronary heart disease; MI: myocardial infarction; **P*<0.05; ***P*<0.01; Multivariate adjusted Logistic regression models of EPA intake with cardiovascular disease in women with dyslipidemia: Model 1: Age and race; Model 2: Model 1+Education, Marital status, Ratio of family income to poverty, BMI, Physical activity, Smoking status and Alcohol consumption； Model 3： Model 2+Hypertension, diabetes, cancer, osteoarthritis, total energy intake, total fat intake, and total cholesterol intake.

Supplemental Table 20

^1^HR: hazard ratio, CI: confidence interval; CVD: CVD mortality; ALL-cause: All-cause mortality; **P*<0.05; ***P*<0.01; Multivariate adjusted Cox proportional regression models of marine polyunsaturated fatty acids intake with cardiovascular disease in women with dyslipidemia: Model 1: Age and race; Model 2：Model 1+education, marital status, ratio of family income to poverty, BMI, physical activity, smoking status and alcohol consumption；Model 3：Model 2+hypertension, diabetes, cancer, osteoarthritis, total energy intake, total fat intake, and total cholesterol intake.

Supplemental Table 21

^1^OR: odds ratio, CI: confidence interval; CHF: congestive heart failure; CHD: coronary heart disease; MI: myocardial infarction; **P*<0.05; ***P*<0.01; Multivariate adjusted Logistic regression models of DPA intake with cardiovascular disease in all male group: Model 1: Age and race; Model 2: Model 1+Education, Marital status, Ratio of family income to poverty, BMI, Physical activity, Smoking status and Alcohol consumption； Model 3： Model 2+Hypertension, diabetes, cancer, osteoarthritis, total energy intake, total fat intake, and total cholesterol intake.

Supplemental Table 22

^1^OR: odds ratio, CI: confidence interval; CHF: congestive heart failure; CHD: coronary heart disease; MI: myocardial infarction; **P*<0.05; Multivariate adjusted Logistic regression models of DHA intake with cardiovascular disease in all male group: Model 1: Age and race; Model 2: Model 1+Education, Marital status, Ratio of family income to poverty, BMI, Physical activity, Smoking status and Alcohol consumption； Model 3： Model 2+Hypertension, diabetes, cancer, osteoarthritis, total energy intake, total fat intake, and total cholesterol intake.

Supplemental Table 23

^1^OR: odds ratio, CI: confidence interval; CHF: congestive heart failure; CHD: coronary heart disease; MI: myocardial infarction; **P*<0.05; ***P*<0.01; Multivariate adjusted Logistic regression models of EPA intake with cardiovascular disease in all male group: Model 1: Age and race; Model 2: Model 1+Education, Marital status, Ratio of family income to poverty, BMI, Physical activity, Smoking status and Alcohol consumption； Model 3： Model 2+Hypertension, diabetes, cancer, osteoarthritis, total energy intake, total fat intake, and total cholesterol intake.

Supplemental Table 24

^1^HR: hazard ratio, CI: confidence interval; CVD: CVD mortality; ALL-cause: All-cause mortality; **P*<0.05; ***P*<0.01; Multivariate adjusted Cox proportional regression models of marine polyunsaturated fatty acids intake with cardiovascular disease in all male group: Model 1: Age and race; Model 2：Model 1+education, marital status, ratio of family income to poverty, BMI, physical activity, smoking status and alcohol consumption；Model 3：Model 2+hypertension, diabetes, cancer, osteoarthritis, total energy intake, total fat intake, and total cholesterol intake.
